# Novel histopathologic feature identified through image analysis augments stage II colorectal cancer clinical reporting

**DOI:** 10.18632/oncotarget.10053

**Published:** 2016-06-15

**Authors:** Peter D. Caie, Ying Zhou, Arran K. Turnbull, Anca Oniscu, David J. Harrison

**Affiliations:** ^1^ Quantitative and Digital Pathology, School of Medicine, University of St Andrews, St Andrews, KY16 9TF, UK; ^2^ Digital Pathology Unit, Laboratory Medicine, Royal Infirmary of Edinburgh, Edinburgh, EH16 4SA, UK; ^3^ Institute of Genetics and Molecular Medicine, University of Edinburgh, Western General Hospital, Edinburgh, EH4 2XU, UK

**Keywords:** digital pathology, big-data, tumor buds, poorly differentiated clusters, data mining

## Abstract

A number of candidate histopathologic factors show promise in identifying stage II colorectal cancer (CRC) patients at a high risk of disease-specific death, however they can suffer from low reproducibility and none have replaced classical pathologic staging. We developed an image analysis algorithm which standardized the quantification of specific histopathologic features and exported a multi-parametric feature-set captured without bias. The image analysis algorithm was executed across a training set (*n* = 50) and the resultant big data was distilled through decision tree modelling to identify the most informative parameters to sub-categorize stage II CRC patients. The most significant, and novel, parameter identified was the ‘sum area of poorly differentiated clusters’ (AreaPDC). This feature was validated across a second cohort of stage II CRC patients (*n* = 134) (HR = 4; 95% CI, 1.5– 11). Finally, the AreaPDC was integrated with the significant features within the clinical pathology report, pT stage and differentiation, into a novel prognostic index (HR = 7.5; 95% CI, 3–18.5) which improved upon current clinical staging (HR = 4.26; 95% CI, 1.7– 10.3). The identification of poorly differentiated clusters as being highly significant in disease progression presents evidence to suggest that these features could be the source of novel targets to decrease the risk of disease specific death.

## INTRODUCTION

Recent reports have demonstrated the potential value of automated image analysis and claimed it as an alternative to conventional clinical histopathologic analysis. The methodology allows the identification and quantification of novel features or the capture of spatial heterogeneity across a tissue section; however neither of which are currently part of routine histopathologic assessment [1, 2]. We have previously shown that automated image analysis complements, rather than replaces, routine histopathology by standardizing the quantification of prognostic histopathologic features in CRC [3]. Unless automated analysis significantly adds to standard practice there will remain a significant barrier to clinical implementation. Here we demonstrate a workflow which utilizes image analysis to extract standardized big data from histopathologic tissue sections and machine learning to distill the most significant resultant parameters addressing a specific clinical question. Furthermore we show how the image analysis based quantification of a tissue sample can be integrated into existing clinical parameters where the aim is to augment rather than replace gold standard practice. The study demonstrates the value of the approach using colorectal cancer as a worked example.

Patients with colorectal cancer (CRC) are staged by the Tumor, Node and Metastasis (TNM) system, although genetic analyses are increasingly a routine part of diagnosis and sub-classification [4–6]. TNM staging is good for prediction of disease progression across the patient population, however it is much less successful at predicting the outcome for individual patients [7]. This is exemplified by the fact that although surgical resection is hoped to be curative for stage II patients, 20% of these patients go on to experience disease-specific death [8]. It is imperative to identify these high risk stage II patients for the inclusion in future clinical trials or to ascertain if detailed follow up could be beneficial [9].

There have been multiple attempts to improve on patient prognosis and to identify novel clinical targets through molecular biomarker analysis and novel histopathologic grading systems, where the invasive margin of CRC is of particular significance [10–12]. Although these features may have prognostic value they have not been adopted into routine clinical practice for several reasons, including observer variability and non-standardized quantification [13–15]; both of which might be overcome using automated image analysis. Furthermore, there could exist unrecognized morphological features containing targetable or prognostic information within the complex and heterogeneous CRC microenvironment. We therefore built an image analysis algorithm which standardized the quantification of a range of candidate prognostic features from the invasive margin of CRC and which have previously shown prognostic significance: tumor budding (TB) [16, 17], poorly differentiated clusters (PDC) [11, 18], lymphatic vessel invasion (LVI) [19], lymphatic vessel density (LVD) [20, 21] and the tumor to stroma ratio [22, 23]. These known features were furthermore compared to other morphometric and spatial parameters captured without bias and measured using hierarchical image analysis.

## RESULTS

### Image analysis and processing workflow

**Figure 1:**
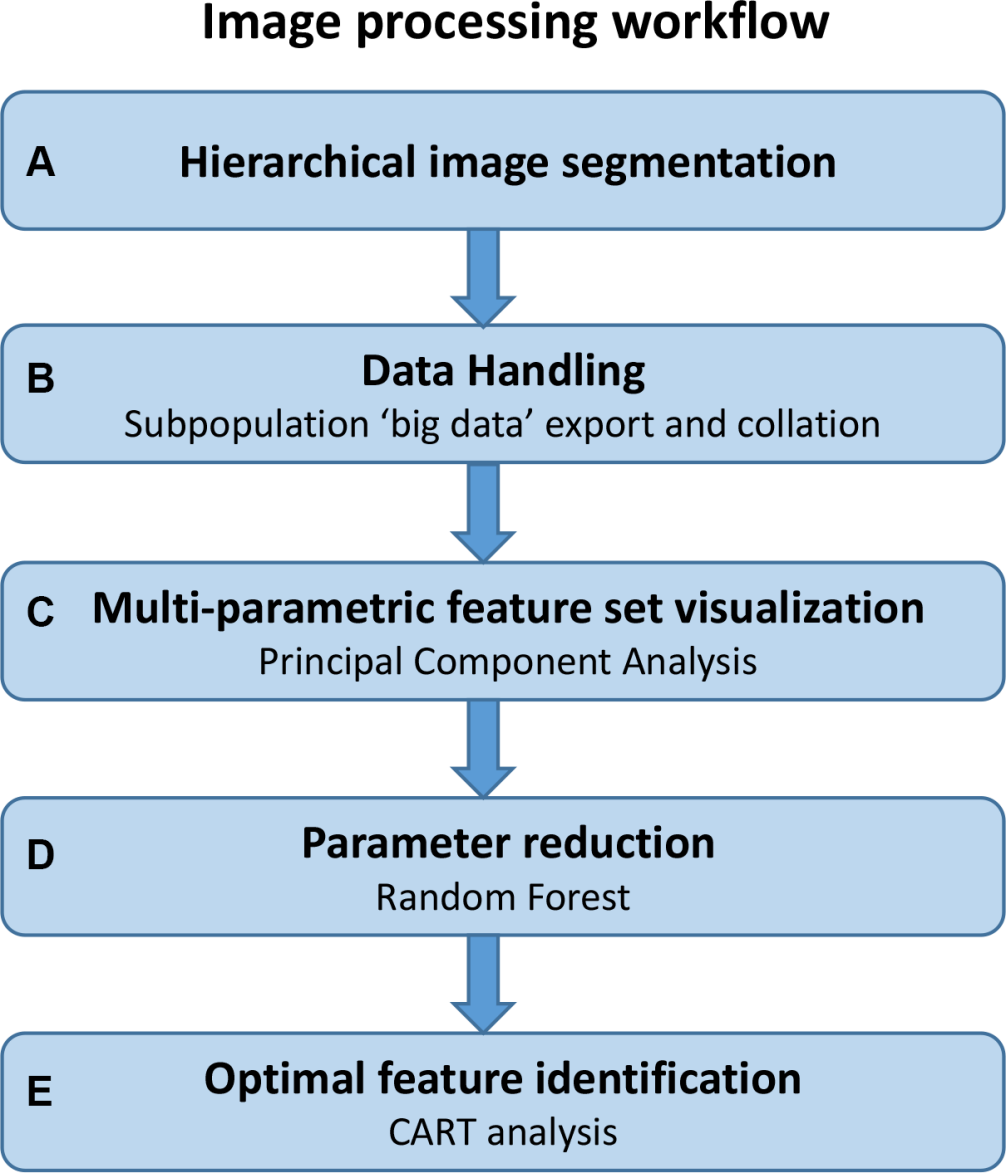
Image processing workflow Steps involved in the imaging process workflow described in this study; from initial image analysis through to the identification of significant parameters by decision tree modelling. This workflow is amenable to any multi-parametric data set where significant parameters and their clinically applicable cut-offs need to be identified.

We have developed a novel image analysis algorithm which quantified candidate histopathologic features in an objective and standardized manner while simultaneously capturing a large, extracted set of unbiased features (123 features in total). The most informative features were subsequently distilled through decision tree modelling. The image processing workflow (Figure 1) was designed to identify the most significant conventional or novel feature-set capable of classifying high or low risk of disease specific death in stage II CRC patients. The workflow was carried out across a training set of 50 patients and the significant parameters identified were validated across a cohort of 134 patients.

### Image segmentation and data export

**Figure 2:**
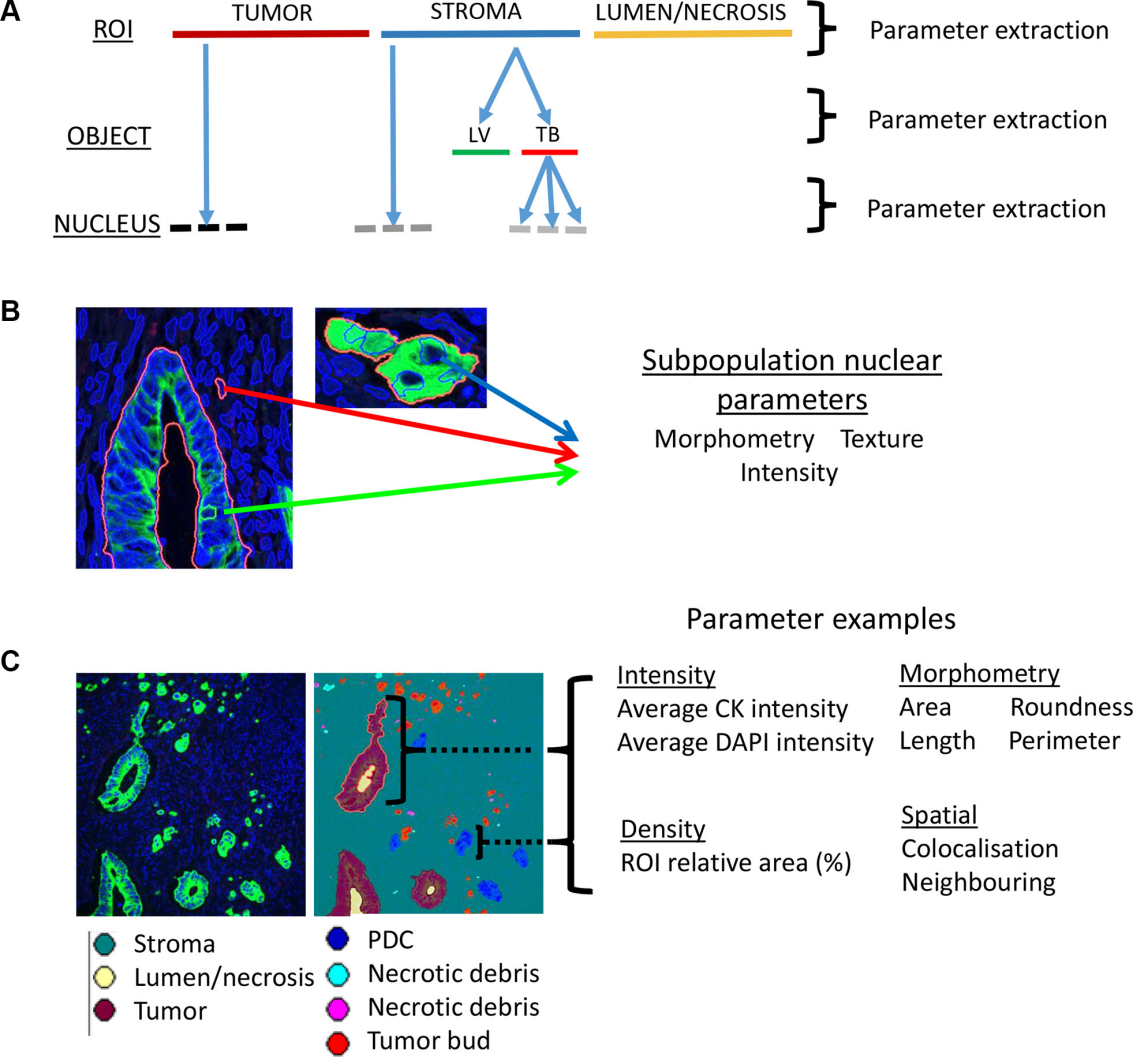
Automated data extraction from analyzed images (**A**) Image based parameters are extracted from each of the hierarchical layers of the image analysis algorithm. The top layer (ROI level) segments tumor from lumen/necrosis and from stroma. The ‘object level’ contains segmented objects such as a lymphatic vessel (LV) or tumor bud (TB) which exist exclusively within an ROI in the layer above e.g. the stroma. The final layer of the hierarchy is the ‘nucleus level’ and contains all segmented nuclei. Nuclei are further segregated into either an object (e.g. tumor) in the ROI level or object (e.g tumor bud) in the object level. The hierarchical approach allows for the capture of heterogeneity across the invasive microenvironment. (**B**) Extraction of parameter classes across nuclei segregated within three distinct heterogeneous objects/ROIs: Tumor gland (nucleus highlighted by green arrow), stroma (nucleus highlighted by red arrow) and tumor bud (nucleus highlighted by blue arrow). (**C**) Visualization of the extraction of different parameter classes and example parameters across the heterogeneous objects (e.g. tumor gland; purple, poorly differentiated cluster (PDC); blue) within a digital tissue image (panCK; green, DAPI; blue).

The algorithm automatically segmented the immunofluorescence labelled digital tissue sections in a hierarchical format (Figure 2A). Immunofluorescence allowed the accurate digital segmentation of stroma, tumor glands (pan cytokeratin (panCK)), invasive tumor subpopulations (panCK), lymphatic vasculature (D2- 40) and all nuclei (DAPI). These objects were sub-classified into candidate histopathologic features prior to quantification: tumor buds, poorly differentiated clusters, lymphatic vessel invasion, minimal lymphatic vessel invasion (less than 5 tumor cells invading vessel), lymphatic vessel density and tumor to stroma ratio. These parameters were specifically included in the algorithm for standardized quantification as they have been shown to have prognostic value but are difficult to accurately reproduce. Furthermore morphometric, density, spatial and fluorescence intensity and texture measurements were captured and exported from the hierarchically segmented objects in an unbiased manner (Figure 2B and 2C). In such a fashion, parameters were extracted from objects across each level of image analysis (Figure 2A). The hierarchical and spatial re-classifying of objects was integrated into the algorithm to capture an aspect of the complex tumor heterogeneity that exists across the invasive front of the CRC microenvironment. The full 123 parameter data-set for each patient sample was subsequently exported and collated (Supplementary Table 1). An extended description of algorithm results and associated Figures is listed in Supplementary Document 1.

### Visualization of the multi-parametric feature-set

**Figure 3:**
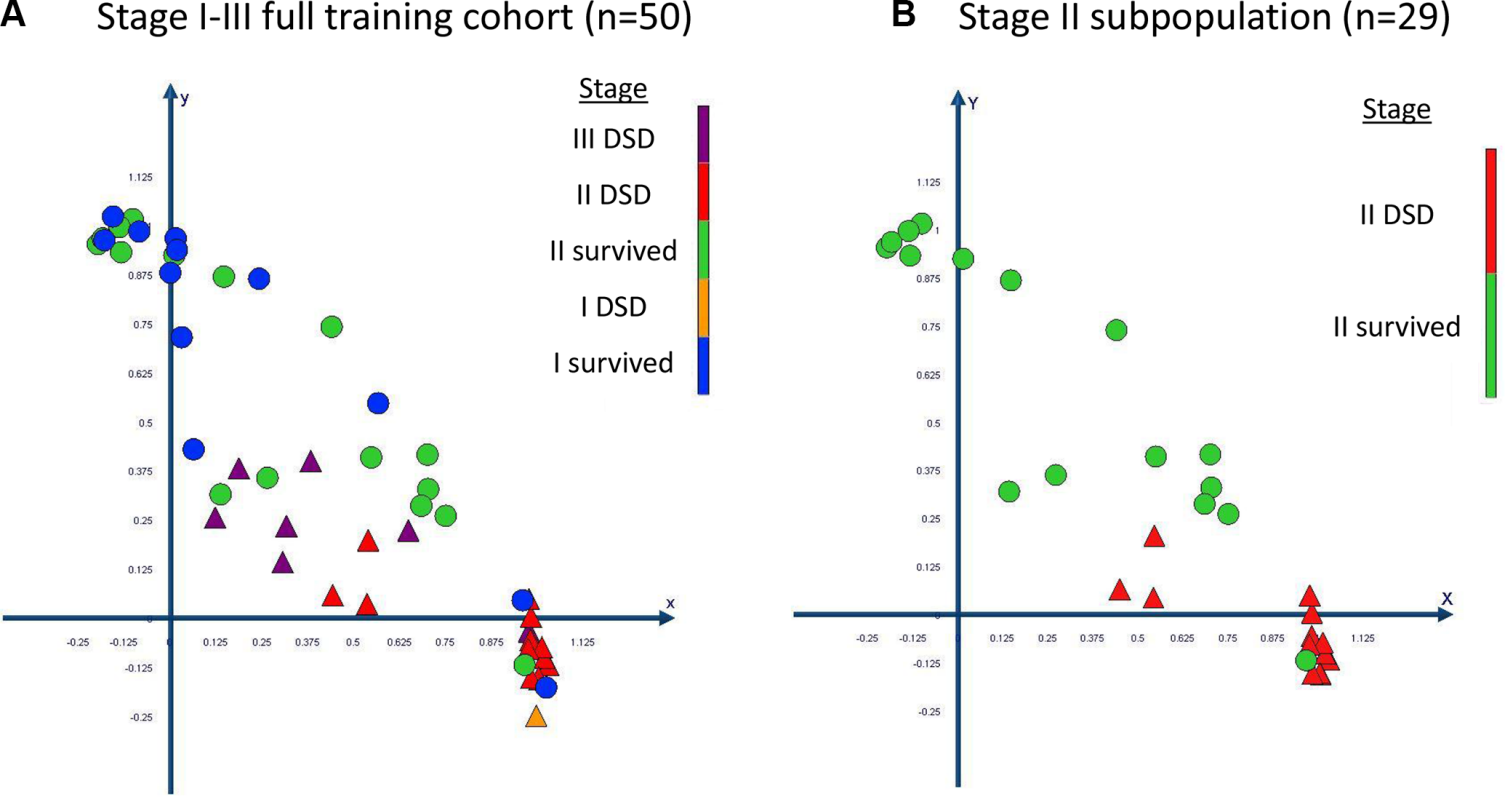
Visualization and clustering of the multi-parametric feature set through principal component analysis (PCA) The data is plotted in a scatter plot for (**A**) the full training set and (**B**) for its stage II subpopulation. Patients who died specifically of CRC are plotted as triangles and patients who survived follow up are plotted as circles. The PCA resulted in the significant categorization of patients at a high and low risk of disease specific death. DSD = disease specific death.

The complex and high dimensional multi-parametric feature-set was visualized in a 2D scatter plot after principal component analysis (PCA) was performed (Figure 3). The 2 plotted principal components explained 72% variance in the full 50 patient training set data. Performing PCA allowed the evaluation of the effectiveness of the full multi-parametric feature-set to categorize patients within the full training cohort into high or low risk of disease specific death. In the stage II subpopulation of the training set there was only one outlier patient who survived follow up and which clustered within the poor outcome group (Figure 3B). The clustering of the full multi-parametric feature-set across the 50 patient training cohort allowed patient categorization on disease specific death with 100% specificity, 78.6% sensitivity and an area under the ROC curve of 0.89. When analyzing the 29 stage II patient subpopulation of the training set, the results were 100% specificity, 93.3% sensitivity and an area under the ROC of 0.94.

### Parameter reduction

Although clustering of the multi-parametric data resulted in the significant categorization of CRC patients on disease specific death, PCA did not report which of the parameters held prognostic significance. To eliminate redundant parameters, while retaining a robust stratification model, we performed random forest analysis.

The 123 parameters within the full multi-parametric feature-set and the full 50 patient training set data were the input for random forest analysis. After analysis the 123 parameters were ranked by their associated Gini score and the least significant parameter was removed. This process was performed iteratively until the removal of a parameter affected the predictive value of the model in a negative manner. The method resulted in a set of 37 parameters (Supplementary Table 1) which had the ability to categorize patients on risk of CRC specific death with the same predictive value as the full multi-parametric feature-set (100% specificity, 76.7% sensitivity, area under the ROC curve of 0.89).

### Novel histopathologic feature identification

Random forest removed 86 redundant parameters from the full multi-parametric feature-set. The remaining 37 significant parameters were used as input to construct a Classification and Regression Tree (CART) model. The CART model exported a single decision tree where parameter relationships and optimal cut-offs for each branch-point within the tree can be reported. CART therefore allows clinically transferable tests to be created from the calculated cut-offs within the optimal parameters reported.

In this study a single parameter was exported within the decision tree as the optimal model. This parameter was the “sum area of poorly differentiated clusters” (AreaPDC) across the invasive front of each tissue section. The inclusion of any further image-based parameters did not, therefore, add to the significance of the model. This single parameter had the ability to significantly categorize the 50 patients from the training cohort by disease specific death with a specificity of 96.3%, a sensitivity of 82.6% and an area under the receiver operator curve (ROC) of 0.9. When the 29 stage II subpopulation of the training cohort was analyzed separately the results were more significant and returned a specificity of 100%, a sensitivity of 93.75% with an area under the ROC of 0.96. The CART model performed 10 fold self-test validation on the full 50 patient training cohort data setwhich reported a specificity of 83.3% and sensitivity of 75% and an area under the ROC of 0.8.

The CART model provided the optimal cut-off from the continuous data captured from the full 50 patient training set across the novel parameter AreaPDC. This cut-off, 35,647 μm^2^, was applied to categorize the patients into groups of high and low risk of disease specific death. Kaplan-Meier curves were subsequently plotted and reported that the AreaPDC was a significant predictor of poor survival and shorter disease specific survival times in both the full 50 patient training cohort (*p* < 0.0001) and its 29 patient stage II subpopulation (*p* < 0.0001) (Supplementary Figure 6). Univariate cox-regression reports that the parameter AreaPDC is a highly significant predictor of disease specific death in the training cohort (HR = 20; 95% CI, 4.6–87.9).

### Validation of image based prognostic features

The analysis algorithm was executed across tissue sections cut from the 134 stage II patient validation set. This was performed primarily to validate the novel prognostic parameter of AreaPDC which we identified in this study. Secondly to compare the prognostic significance of the image analysis quantified set of candidate histopathologic features and the novel parameter.

The validation set patients were separated into categorical groups of either above or below the training set cut-offs for each parameter. Optimal validated cut-offs and their prognostic significance for the training set are listed in Supplementary Table 2. Disease specific death statistics were calculated as univariate Cox-regression and Kaplan-Meier analysis for the validation set data. Univariate Cox-regression reported that tumor budding (HR = 2.5; 95% CI, 1–6), minimal lymphatic vessel invasion (HR = 2.5; 95% CI, 1–6), poorly differentiated clusters (HR = 3; 95% CI, 1.2–9), tumor to stroma ratio (HR = 0.1; 95% CI, 0.03–0.6) and the AreaPDC (HR = 4; 95% CI, 1.5– 11) were significant predictors of disease specific death within the validation set whereas lymphatic vessel density and lymphatic vessel invasion were not (Table 1). The novel parameter AreaPDC returned a higher associated significance (*p* = 0.007) than any of the candidate histopathologic parameters apart from the tumor to stroma ratio which reported the same significance (*p* = 0.007). No single parameter identified through image analysis had a higher prognostic significance than the clinically reported pT stage (HR = 4.26; 95% CI, 1.76–10.33, *p* = 0.001).

**Table 1:** Validation set patient data

Clinicopathologic parameters	patient number (*n*)	Univariate			
		HR	95% CI		*P* value
			Lower	Upper	
**Clinical pathology report**					
**Stage**					
II	134				
**Gender**		0.93	0.6	1.45	0.76
M	69				
F	65				
**Age at Diagnosis**		1.4	0.82	2.41	0.22
≤ 70	52				
71–79	39				
≥ 80	43				
**pT Stage**		**4.26**	**1.76**	**10.33**	**0.001**
pT3	102				
pT4	32				
**Differentiation**		**2.17**	**1.14**	**4.13**	**0.018**
Well	3				
Moderate	109				
Poor	22				
**Histology**		0.82	0.27	2.49	0.72
AC	121				
MC	7				
AC/MC	6				
**Site**		0.87	0.5	1.51	0.61
Rectal	**42**				
Right side	**47**				
Left side	**45**				
Tumor Diameter*		1.7	0.66	4.42	0.27
High	**44**				
Low	**90**				
**Total Node Examined**		0.6	0.24	1.5	0.27
< 12	35				
≥ 12	99				
**EM LVI**		**2.8**	**1.1**	**7.3**	**0.04**
Yes	**20**				
No	**114**				
**Image Analysis**					
**Tumor Budding**		**2.49**	**1.03**	**5.99**	**0.04**
High	44				
Low	90				
**Minimal LVI**		**2.46**	**1**	**6.05**	**0.05**
High	35				
Low	99				
**LVI**		1.9	0.75	5.15	0.16
High	**27**				
Low	**107**				
**Tumor to stroma ratio**		**0.13**	**0.03**	**0.57**	**0.007**
High	76				
Low	58				
**LVD**		1.39	0.46	4.16	0.56
High	25				
Low	109				
**Number PDC**		**3.03**	**1.08**	**8.5**	**0.04**
High	18				
Low	116				
**Area PDC**		**4.02**	**1.46**	**11.1**	**0.007**
High	65				
Low	69				
**NPI**		7.5	3	18.5	0.00001
High	22				
Low	112				

Patient data was extracted from the clinical pathology report and categorized prior to univariate Cox-regression (top half of table). Image analysis data was categorized into above or below training set cut-off (bottom half of table) prior to univariate Cox-regression. Novel Prognostic Index (NPI) of integrated Area PDC, pT stage and differentiation is bordered by double line. HR = hazard ratio, CI = confidence interval. EM LVI = Extramural lymphovascular invasion, LVI = lymphatic vessel invasion, LVD = lymphatic vessel density, PDC = poorly differentiated clusters. *No entry in pathology report for tumor diameter in 18 cases. Parameters in bold were significant; significance was set at (*p* ≤ 0.05).

**Figure 4:**
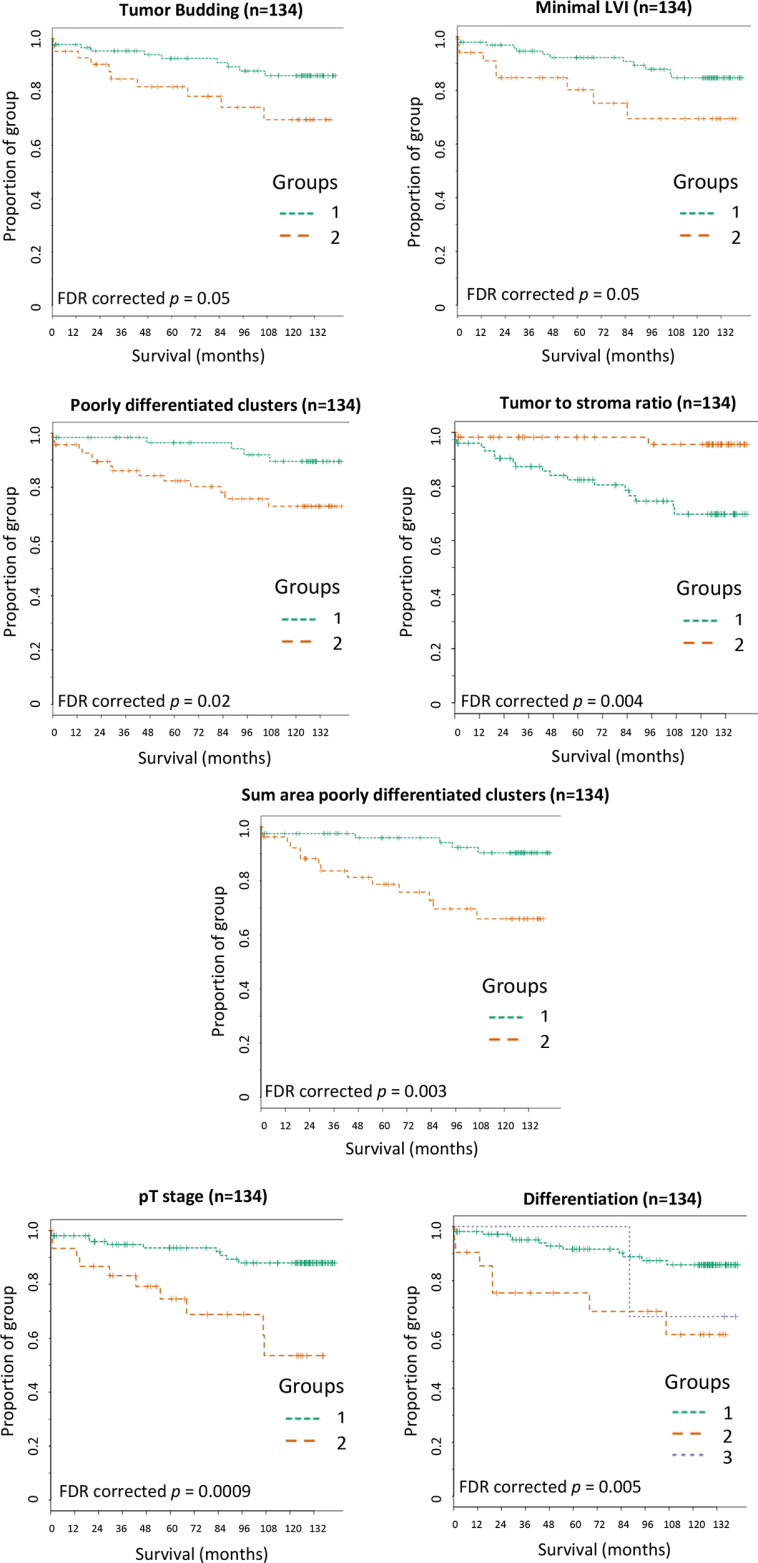
Kaplan-Meier survival curves for the significant histopathologic parameters captured across the validation set Patients within group 1 were below the training set cut-off for the associated parameter and patients within group 2 were above cut-off. (**A**) Tumor budding: training set cut-off of 287 buds. (**B**) Minimal LVI: training set cut-off of 16 events. (**C**) Poorly differentiated clusters: training set cut-off of 35 clusters. (**D**) Tumor to stroma ratio: training set cut-off of 21% tumor area of the total tissue. (**E**) Sum area poorly differentiated clusters (AreaPDC): training set cut-off of 35647 μm^2^. (**F**) pT stage: pT3 = group1 and pT4 = group 2. (**G**) Differentiation: Well = group 1, Moderate = group 2 and Poor = group 3. FDR = false discovery rate corrected *p* values.

Kaplan-Meier curves were plotted for the significant parameters utilizing the categorized validation cohort data to assess disease specific survival over time (Figure 4). Patients in the high cut-off group for tumor budding (*p* = 0.05), poorly differentiated clusters (*p* = 0.02), minimal lymphatic vessel invasion (*p* = 0.05), AreaPDC (*p* = 0.003) as well as patients in the lower cut-off group for the tumor to stroma ratio (*p* = 0.004) had a higher risk of disease specific death and significantly shorter survival times than patients in the alternative group. The Kaplan-Meier analysis confirmed the novel parameter of AreaPDC to hold the highest significance of the risk of disease specific death over time than any of the other histopathologic parameters, although this was less than clinical pT staging (*p* = 0.0009).

### Novel prognostic index augments clinical staging

The significant parameters reported by univariate cox-regression (Table 1), from either image analysis or the clinical pathology report, were entered into a multivariate backward elimination Cox-regression model. This was performed to identify which parameters added significant value to an integrative model in its ability to predict CRC specific death within the validation set. The integration of AreaPDC (*p* = 0.02), T stage (*p* = 0.03) and differentiation (*p* = 0.04) increased the significance of a final predictive model. The image analysis parameters of tumor budding (*p* = 0.8), poorly differentiated clusters (*p* = 0.51), minimal lymphatic vessel invasion (*p* = 0.8) and the tumor to stroma ratio (*p* = 0.55) or the clinical parameter of extra-mural lymphovascular invasion (EM LVI) (*p* = 0.18) did not add further significance to the model and were therefore excluded (Table 2A).

**Table 2A:** Parameters entered into the backwards elimination Cox Regression model

Variables in the equation	Multivariate Cox Regression Model (Ordinal Variables)			
	HR	95% CI		*P* value
		Lower	Upper	
Area PDC	3.3	1.1	9.3	0.005
pT stage	2.9	1.1	7.3	0.030
Differentiation	2.2	1	4.9	0.046
**Variables not in the equation**				
EMLVI				NS
Tumor to stroma ratio				NS
TB				NS
PDC				NS
Minimal LVI				NS

Variables which add significance (top half of table) and variables which do not add significance (bottom half of table) to an integrative model to predict disease specific death. NS = Non-significant. Area PDC = area of poorly differentiated clusters, EMLVI = extramural lymphovascular invasion, TB = tumor budding, PDC = poorly differentiated clusters, Minimal LVI = minimal lymphatic vessel invasion.

The three significant parameters within the forward conditional model were compiled into a novel prognostic index. Patients with an above cut-off score in two or more of the three significant parameters were classified as a “high-risk” of disease specific death group and the remainder of the patients were classified with a “low-risk” score. The novel prognostic index was the sole significant parameter to predict disease specific death (HR = 7.5; 95% CI, 3–18.5, *p* = 0.00001) (Table 1) when entered into a backward elimination Cox regression model along with its composite parts (Table 2B). The novel prognostic index's prediction of CRC specific death reported a significant improvement on classical pT staging (HR = 4.26; 95% CI, 1.76–10.33, *p* = 0.001) within this stage II CRC patient validation cohort.

**Table 2B:** Parameters entered into the backwards elimination Cox Regression model

Variables in the equation	Multivariate Cox Regression Model (Ordinal Variables)			
	HR	95% CI		*P* value
		Lower	Upper	
NPI	7.5	3	18.4	0.00001
**Variables not in the equation**				
Area PDC				NS
pT stage				NS
Differentiation				NS

Variables which add significance (top half of table) and variables which do not add significance (bottom half of table) to an integrative model to predict disease specific death. NS = Non-significant. Area PDC = area of poorly differentiated clusters.

**Figure 5:**
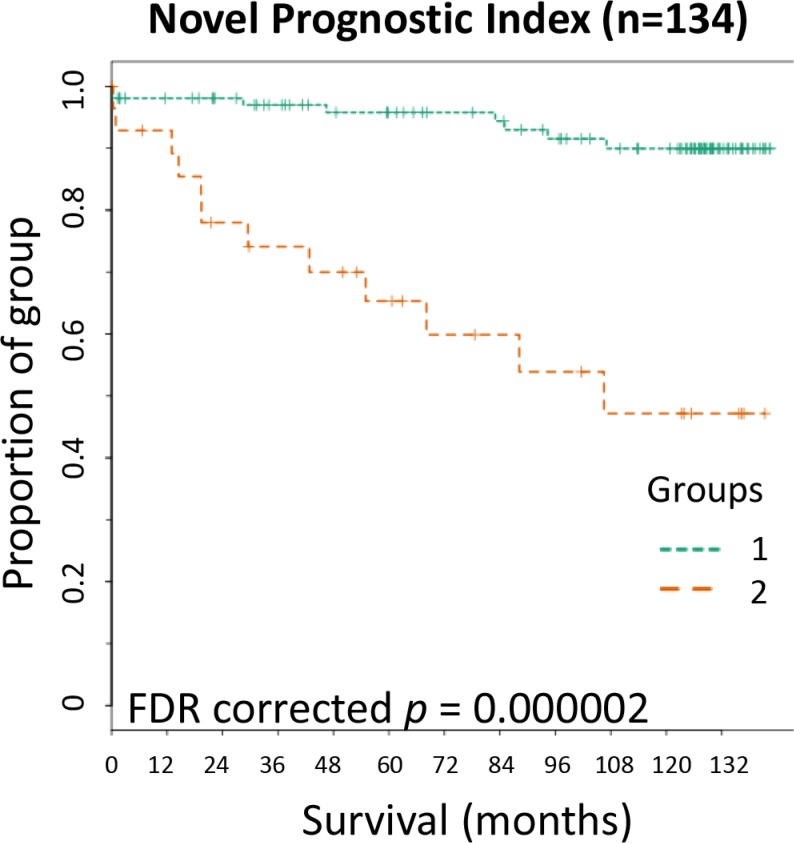
Kaplan-Meier survival curve for the Novel Prognostic Index Novel Prognostic Index; patients within group 2 were within above cut-off in two or more of the composite parameters while those in group 1 were within above cut-off of one or no composite parameters. FDR = false discovery rate corrected *p* values.

Kaplan-Meier curves were plotted to assess the significance of the novel prognostic index in patient survival over time (Figure 5). The novel prognostic index significantly categorized stage II CRC patients with a high risk of CRC specific death over an 11.5 year follow up (*p* < 0.0001).

## DISCUSSION

We have demonstrated that a novel feature, area of poorly differentiated clusters (AreaPDC) identified through image analysis can augment traditional histopathologic staging of stage II CRC, moving toward a more personalized prognosis. Furthermore the evidence that the involvement of poorly differentiated clusters is highly significant in disease progression directs future research into the elucidation of their molecular phenotype and thereby identifying novel targets to inhibit their invasive characteristics. The novel feature (AreaPDC) was distilled through decision tree modelling from a large hierarchical and multi-parametric feature-set which contained both candidate histopathologic features and ones captured in an unbiased manner. The data was entered into the model with no knowledge *a priori* of which image based parameter would hold clinical significance. The multi-parametric feature-set itself was captured through a novel image analysis algorithm developed within this study and which was executed across digitized CRC tissue sections from a training and validation cohort. The patients within the validation cohort were significantly categorized into high and low risk of disease specific death (HR = 4; 95% CI, 1.5–11), depending on their sum area of poorly differentiated clusters (AreaPDC) being above or below the cut-off of 35647 μm^2^. AreaPDC was more informative than previously reported prognostic features which were simultaneously captured across each patient sample through the bespoke image analysis algorithm, including number of tumor budding [16, 17] number of poorly differentiated clusters [11, 18], tumor to stroma ratio [22, 23] and lymphatic vessel invasion [19]. Although these previous studies reported prognostic significance in CRC as individual parameters, none have simultaneously co-registered all of the quantified features across a single tissue section. We have demonstrated here that tumor budding, poorly differentiated clusters, tumor to stroma ratio and lymphatic vessel invasion also hold univariate significance in the prediction of disease specific death but that they become redundant and add no further independent valuewhen integrated into a multi-variable model which includes the AreaPDC. Furthermore predictive significance increased upon the creation of a novel prognostic index where AreaPDC was integrated with the significant clinical data parameters of differentiation and pT stage (HR = 7.5; 95% CI, 3–18.5, *p* = 0.00001). Although the use of continuous data is optimal within regression models [24], translatable tests, at least for the foreseeable future, rely on a validated cut-point to inform on clinical decision making. The novel feature AreaPDC was therefore dichotomized using the validated CART reported cut-off prior to entering into regression models which included pre-existing categorical clinical data (pT stage and differentiation). Therefore the potentially translatable novel prognostic index predicts disease specific death in a stage II CRC validation cohort with more accuracy than the standalone conventional pT staging, however further validation of this index will be required.

Previous studies have demonstrated the advantages of image analysis reported large data sets captured in an unbiased manner [1] or to quantify set histopathologic features [3, 25]. Uniquely, the image analysis algorithm developed in this study captured a combination of both in a standardized and objective manner and demonstrated the validity in principle of such methodology. The quantified data was captured in a continuous format which allowed optimal cut-offs for parameters to be calculated and subsequently validated. The multi-parametric feature-set was analyzed in two separate decision tree models. Random forest was utilized to negate redundant parameters which added no value to the predictive binary outcome of disease specific death. A Random forest model [26], consisting of 5000 trees, ranked and reduced the parameters according to their Gini score and significance in the model and is an ideal model for handling large data sets. It is not applicable to a clinical prognostic index as it does not inform on cut-offs or the combinations of parameters which yield the optimal output to stratify patients. A CART model [27], which produces a single decision tree and works more accurately on smaller data sets, was subsequently run across the significant parameters identified through random forest. CART provides optimal and validated cut-offs for each parameter at each branch point within a model and is therefore amenable to the identification of a clinically transferable histopathologic marker or a combination of markers.

The semi-quantification of current and emerging histopathologic features in CRC may suffer from poor reproducibility and inter- and intra-observer variability [10, 14, 15]. Previous studies in tumor budding and poorly differentiated clusters have concentrated on their number [16–18, 28] and not their area; which would prove difficult to accurately report by eye. Image analysis offers the standardization and the fully reproducible quantification of features, such as the accurate reporting of the area of poorly differentiated clusters (AreaPDC). This study employed immunofluorescence for the multiplexing, co-registering and accurate quantification of the features captured across the CRC tissue section. Lymphatic vessel invasion and mesenchymal transitioned invading cancer cells may prove particularly obscure under classical H&E stained tissue. We acknowledge that although advantageous for initial investigative purposes, immunofluorescence may not be applicable to routine clinical use. However, the single novel parameter AreaPDC can be quantified through chromogenic immunohistochemistry using a wide spectrum cytokeratin antibody and a much simplified image analysis algorithm making it amenable to most clinical laboratories. This translational impact would still rely on the wider adoption of digital pathology in routine clinical practice but which is predicted in the near future [29]. Although there are molecular tests to predict poor outcome in stage II CRC patients [30] they tend to homogenize tissue which destroys spatial heterogeneity. Image analysis may unlock a wealth of valuable histopathologic information form the heterogeneous tumor microenvironment, while retaining the spatial resolution of interacting host and tumor subpopulations. In time this can be integrated with molecular genetic data [31]. The area of poorly differentiated clusters, identified as a distinct subpopulation, were the most significant predictor of disease specific death and exemplifies heterogeneous data which may be lost through molecular testing.

Image analysis across tissue microarrays (TMAs) yielded novel prognostic features in breast cancer although the authors acknowledged that analyzing whole slide images could report a more significant model [1]. We found that quantifying the candidate histopathologic features across a single core of a TMA per patient yielded insignificant results (data not shown). We therefore adopted a whole slide imaging approach which allowed the identification of the invasive front of the tumor section and the capture of high resolution images from within this region. We concentrated our analysis on the invasive front as the candidate histopathologic features of interest are predominantly located in, and conventionally reported from, this region. The data captured across these images allowed the significant categorization of high risk of disease specific death CRC patients. Further investigation would need to be performed to identify the optimal number of images required to be captured across the whole section in order to yield reproducible results across potential further validation cohorts.

We utilized proprietary software for the image analysis and data mining aspects of this study. We endeavored to ensure that the overall workflow presented here for novel feature identification from multi-parametric data is possible within open source software thus making it amenable to most clinical research labs. Both random forest and CART can be executed within the statistical package R (http://www.statmethods.net/advstats/cart.html) while image analysis based multi-parametric feature extraction can be performed in Image J (http://imagej.nih.gov/ij/) or Cell Profiler (http://www.cellprofiler.org/). Similarly the workflow is not limited to the specific immunofluorescence assay, candidate histopathologic features or multi-parametric feature set utilized within this study but can be applied across any digital pathology specimen.

TNM staging aided by the core clinical data-set [8, 32] has long remained the gold-standard for the prediction of CRC disease progression. Although, there exists debate on how TNM staging can be improved upon while retaining it as central to CRC prognosis [33]. Therefore it would seem pertinent to include the pathological reporting which already exists into novel integrative models. The novel prognostic index reported here returned a higher significance of high-risk patient identification than the sum of its parts. This study therefore demonstrates the value of systematic reporting incorporated into a novel prognostic index over the reporting of a single feature in isolation. Although image analysis will unlikely completely replace conventional histopathology it can be applied to standardize the quantification of certain histopathologic features and play a vital role in identifying novel prognostic and targetable parameters, such as poorly differentiated clusters, amenable to clinical translation and integration in order to address urgent clinical needs.

## MATERIALS AND METHODS

### Patient samples

Tissue samples were residual diagnostic tissue stored within the NHS Lothian Department of Pathology archive. The research was undertaken under the approval of the NHS Lothian NRS BioResource and ethical approval for the study (13/ES/0126) was granted by the East of Scotland Research Ethics Service.

Training set (*n* = 50): Twenty nine stage II patient samples were selected on colorectal specific death (15 survived follow up: good outcome and 14 died of disease: poor outcome) from a Scottish prospectively collected CRC cohort. Patient follow up was for at least 15 years. Thirteen stage I (12 with good outcome and 1 with poor outcome) and 8 stage III (all of whom died of disease) patients were randomly selected from the cohort for comparison. Patient samples were collected, after surgical resection, between the years of 1996 and 2003, from hospitals across Scotland. No patients within the training cohort underwent neoadjuvant or adjuvant therapy. Patient data is listed in Supplementary Table 2.

Validation set (*n* = 134): A separate validation set comprised all cases of stage II CRC treated in Edinburgh (Scotland) hospitals over 2 concurrent years (years 2002 and 2003), resulting in a cohort of 147 patients. Clinical follow up was up to 11.5 years. Due to insufficient available material 13 patients were excluded leaving a remaining 134 patients within the validation set; 20 of whom died of disease during the follow up period. Of the 134 patients tested within the validation set 9 rectal cancer patients underwent adjuvant therapy and 3 colon cancer patients under went adjuvant chemotherapy. Patient data is listed in Table 1.

### Immunofluorescence and image capture

Samples were prepared for multiplexed immunofluorescence with DAPI (nuclei) and antibodies against pan-cytokeratin (epithelial cells) and D2-40 (lymphatic endothelium), as previously outlined [3]. Briefly, a 4 μm section was cut from a FFPE tissue block, deparaffinised and rehydrated. Microwave based antigen retrieval (Tris-EDTA, pH9 buffer), endogenous peroxidase (3% H_2_0_2_) and protein block (Dako, X0909) steps were undertaken prior to immunofluorescence. Immunofluorescence was performed using antibodies against wide specificity cytokeratin (pan-cytokeratin) (primary antibody: Dako, Z0622, 1:150; Alexa Fluor 555 secondary antibody: Thermo Fisher Scientific, A21428, 1:25) and lymphatic endothelium (D2-40, primary antibody: Dako, M3619, 1:2000; HRP labelled secondary antibody: Dako, K4001 and Cy5 Tyramide, Perkin Elmer, SAT705A001EA, 1:100). Tissue sections were counterstained in DAPI Prolong Anti-fade mountant (Thermo Fisher Scientific, P36931).

All images were captured on the HistoRx PM-2000 imaging platform (HistoRx Ltd., Branford, CT, USA). Whole slide images were captured with a 4x objective through the pan-cytokeratin (Alexa Fluor 555/Cy3) channel. For each tissue section, the invasive front was identified manually from the whole slide image and 15 high-resolution images were captured, in an evenly spaced distribution across the invasive front, through a 20x objective, with the following settings: DAPI (200 ms exposure), Cy3 (35 ms exposure) and Cy5 (200 ms exposure), thus visualizing nuclei, pan-cytokeratin (panCK) and lymphatics with D2-40 labelling respectively. All patient samples were treated in a standardized manner as described above. Images are exported from the PM- 2000 platform as .TIFF files at highest resolution. Each field of view is exported as three separate greyscale. TIFF files; one for each associated wavelength.

### Image analysis

The novel image analysis algorithm was created within the Definiens image analysis software packages: Tissue Studio^®^ and Developer XD™ (Definiens AG, Munich). Images were imported into Definiens as .TIFF files. Initial image segmentation utilized an image analysis algorithm created in Tissue Studio^®^ as described previously [3]. All segmented objects were further classified within a hierarchical system where the top level was automatically segmented through machine-based learning using Definiens Composer Technology™ into Regions of Interest (ROIs): ‘tumor’, ‘necrosis/lumen’, ‘no tissue’ and ‘stroma’. Next, the object level of the image analysis hierarchy captured all panCK and D2-40 positive objects in the stroma. The final layer of image analysis identified nuclei through the DAPI channel. Each nucleus was exclusively segregated into relevant subpopulations existing in the analysis layers above. The Tissue Studio^®^ analyzed workspace was subsequently imported into Developer XD™ for bespoke object classification, optimization and parameter export. A full description of the image analysis methods, with accompanying figures and settings for the Definiens’ rulesets are listed in Supplementary Document 2.

### Multi-parametric data extraction

The algorithm quantified the total number of objects within each classification as well as extracting morphometric, spatial relationships, texture and fluorescence measurements from the objects across the segmented hierarchical image layers. The parameters captured from each of the 15 images taken per tissue section were either averaged or summed (depending on the nature of the parameter) to equate to one data-point per parameter per patient. In total 123 image-based parameters were extracted per tissue section and these made up the multi-parametric feature-set for each patient sample. A table containing the full list of the extracted parameters, and their collation method per patient sample, is located in Supplementary Table 1.

### Statistical analysis

#### Optimal training set cut-offs

The unprocessed, continuous data for each candidate histopathologic feature (tumor budding, poorly differentiated clusters, lymphatic vessel invasion, minimal lymphatic vessel invasion, tumor to stroma ratio and lymphatic vessel density) alongside patient outcome data was loaded into X-Tile software [34] to calculate the optimal cut-offs to categorize patients into high or low risk of disease-specific death. The significance of these cut-offs were corrected by cross-validation within Monte-Carlo simulations (*n* = 1000). Training set cut-offs were subsequently applied to the validation set to categorize the stage II patient population.

#### Modelling to identify significant parameters

Principle Component Analysis and Cox regression calculations (univariate and multivariate backwards elimination with a stopping rule based on Aikaike's Information Criteria) were performed in SPSS (IBM, New York, USA). From the 123 multi-parametric feature set the most informative features which differentiated between binary disease specific survival were identified by inputting the continuous data for each parameter into a random forest (*n* = 5000) decision tree model [26] and exporting the associated Gini score. The continuous data from each informative parameter was subsequently input into a classification and regression tree (CART) [27] decision tree strategy to identify optimal combinations of and clinical cut-offs of novel significant histopathologic features (Salford Predictive Miner, Salford Systems, San Diego, USA). To avoid over-fitting within the decision tree modelling, validation was performed during the decision tree modelling for both random forest (out of bag) and CART (10 fold self-test). Kaplan Meier curves and associated Benjamini-Hochberg false discovery rate corrected *p* values were calculated using TMA Navigator (http://www.tmanavigator.org) [35].

## SUPPLEMENTARY MATERIALS




