# Supplementary material for: Novel histopathologic feature identified through image analysis augments stage II colorectal cancer clinical reporting
Source: Oncotarget. 2016 Jun 15;7(28):44381–94. doi: 10.18632/oncotarget.10053 (PMC5190104; doi:10.18632/oncotarget.10053)
Supplement: Supplementary file 1 [file oncotarget-07-44381-s001.pdf]

Novel histopathologic feature identified through image analysis augments stage II colorectal cancer clinical reporting

Supplementary Materials

SUPPLEMENTARY DOCUMENT 1

Extended results and figures for the image analysis algorithm

The algorithm automatically segmented the immunofluorescence labelled digital tissue sections in a hierarchical format (Figure 2A). Immunofluorescence allowed the accurate digital segmentation of stroma, tumor glands (panCK), invasive tumor subpopulations (panCK), lymphatic vasculature (D2-40) and all nuclei (DAPI). These objects were sub-classified by the Developer XD™ programmed section of the algorithm into candidate histopathologic features prior to quantification. PanCK objects in the stroma with 5 or less associated nuclei were classified as ‘tumor buds’ and those with more than 5 associated nuclei and which were not gland forming (did not contain automatically identified areas of lumen or necrosis in the center of the cluster of tumor cells) were classified as ‘poorly differentiated clusters’ (Supplementary Figure 1, below). Lymphatic vasculature invasion was arbitrarily split into two sub-categories which we termed: ‘lymphatic vessel invasion’ (where more than 5 tumor cells had invaded the vessel) and ‘minimal lymphatic vessel invasion’ (where 5 or less tumor cells had invaded the vessel) (Supplementary Figure 2, below). Lymphatic vessel density (Supplementary Figure 3, below), nuclei (segregated into 3 distinct subpopulations of stromal nuclei, tumor gland nuclei and tumor bud nuclei (main text Figure 2B)), the tumor to stroma ratio and tumor gland morphometry (Supplementary Figure 4, below) were also quantified and exported. Furthermore morphometric, density, spatial and fluorescence intensity and texture measurements were captured and exported from the hierarchically segmented objects in an unbiased manner (main text Figure 2B and 2C). In such a fashion, parameters were extracted from objects across each level of image analysis: at the top region of interest (ROI) level, object level or nucleus level (main text Figure 2A). Spatial information, such as neighboring and co-localization of objects was also captured and used to further sub-classify; such as vessels bordering tumor glands or tumor buds bordering vessels (Supplementary Figure 5, below). The hierarchical and spatial re-classifying of objects was integrated into the algorithm to capture an aspect of the complex tumor heterogeneity that exists across the invasive front of the CRC microenvironment. The multi-parametric single object data which was exported and subsequently collated ensured a single row of data per patient sample. In total 123 parameters across all levels of image analysis were collated and these parameters comprised the full multi-parametric data-set for each patient sample (Supplementary Table 1). Five of these parameters: tumor budding, poorly differentiated clusters, lymphatic vessel invasion, lymphatic vessel density and tumor to stroma ratio, were specifically included in the algorithm for standardized quantification as previously they have been shown to have prognostic value but are difficult to reproduce between observers.

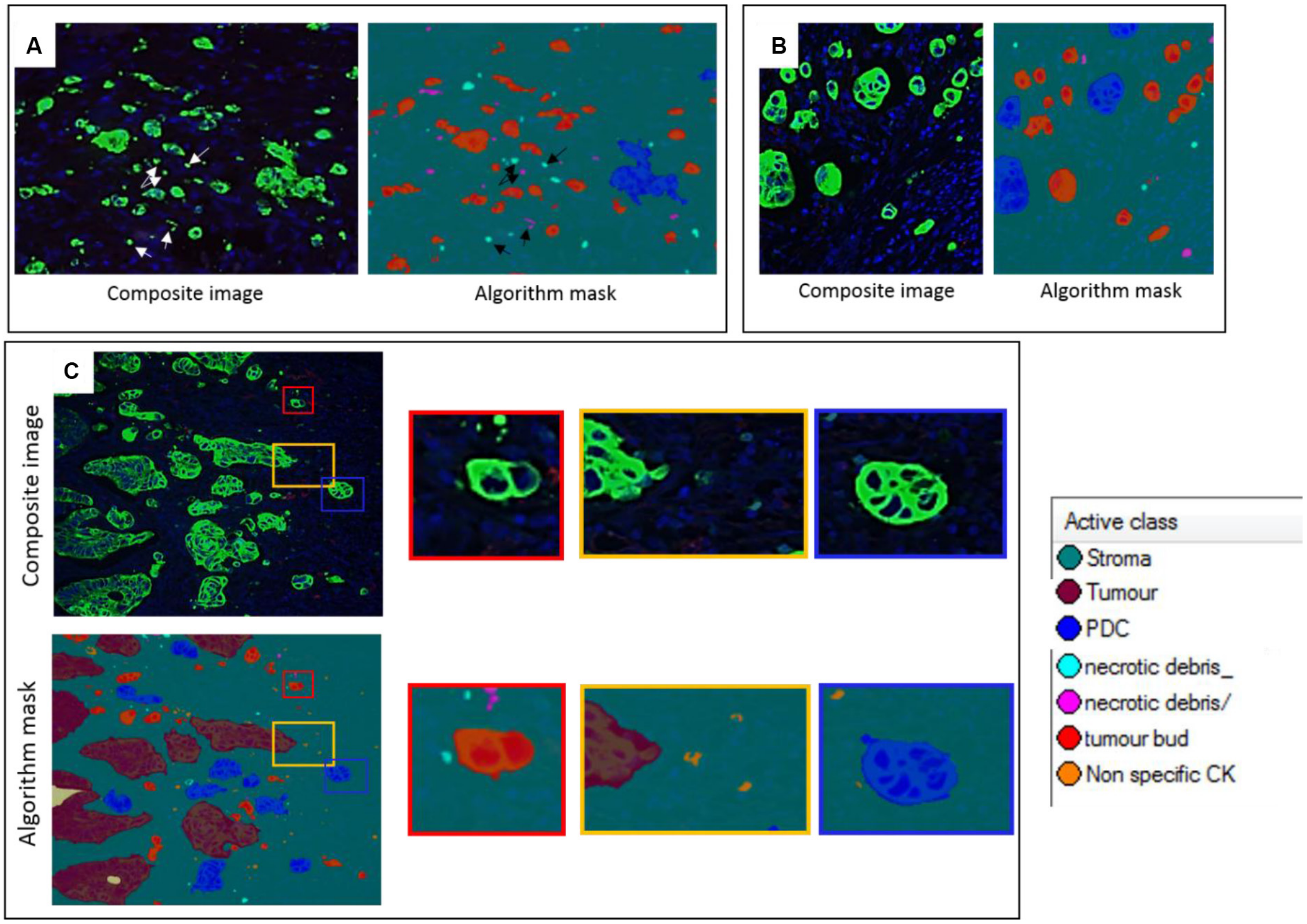

Supplementary Figure S1: Automatic classification of tumor buds and poorly differentiated clusters. (**A**) Composite image of a high budding area at the invasive front of a CRC tissue section (panCK; green, DAPI; blue) and the corresponding algorithm mask classifying: stroma (turquoise) tumor buds (red), poorly differentiated clusters (dark blue) and arrows highlighting necrotic debris (pink or light blue). (**B**) Digital zoom of an area of tumor buds and poorly differentiated clusters and the corresponding classifications by the algorithm. (**C**) An infiltrative invasive pattern showing tumor glands (purple), stroma (turquoise), poorly differentiated clusters (dark blue) and tumor buds (red). Furthermore the highlighted orange square in (C) demonstrates areas of autofluorescence which have been classified as ‘Non specific CK’ (orange mask) and were excluded by the algorithm.

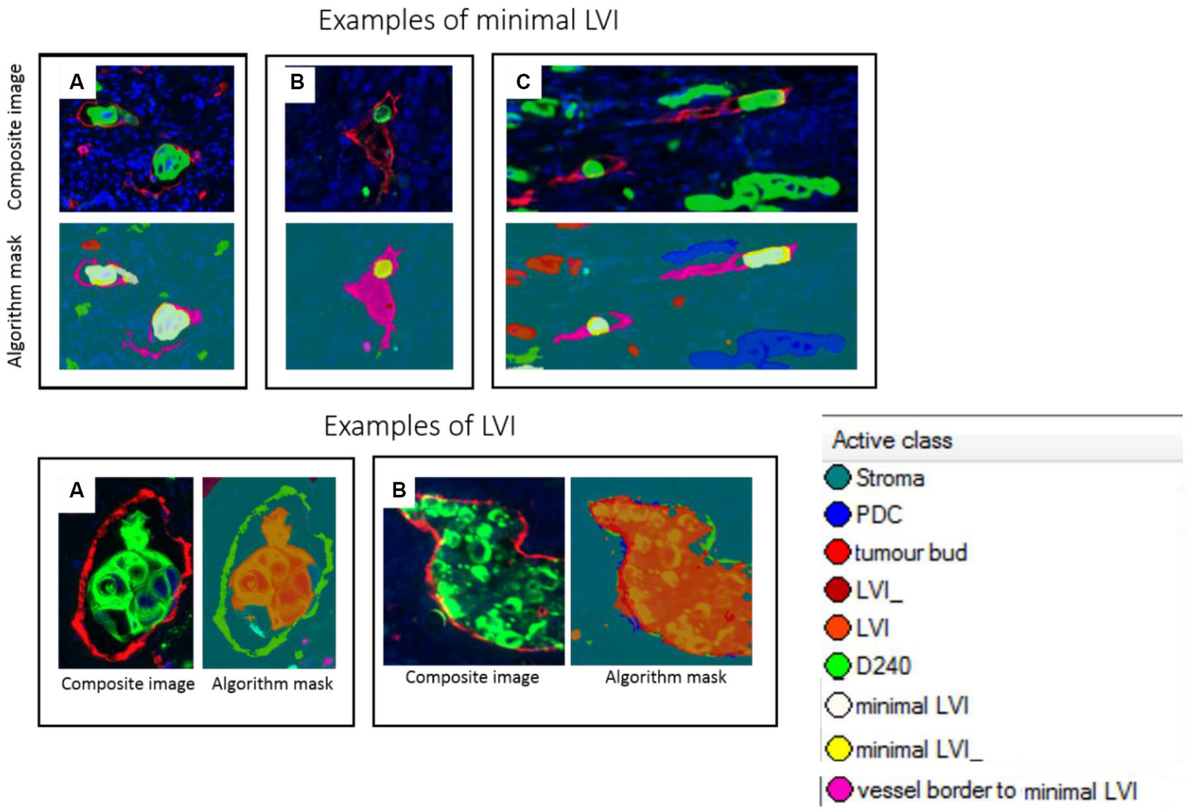

Supplementary Figure S2: Automatic classification of minimal lymphatic vessel invasion (Minimal LVI) (top). (**A, B** and **C**) show examples of minimal lymphatic vessel invasion where 5 or less tumor cells have invaded a lymphatic vessel (composite image: blue; DAPI, green; panCK, red; lymphatic vessel(D2-40)). The algorithm mask identifies numbers and areas of tumor cell invasion (white and yellow) and vessels which have been invaded (pink). Automatic classification of lymphatic vessel invasion (LVI) (bottom). A & B show examples of lymphatic vessel invasion where more than 5 tumor cells have invaded a lymphatic vessel (composite image: blue; DAPI, green; panCK, red; lymphatic vessel(D2-40)). The algorithm mask identifies numbers and areas of tumor cell invasion (orange).

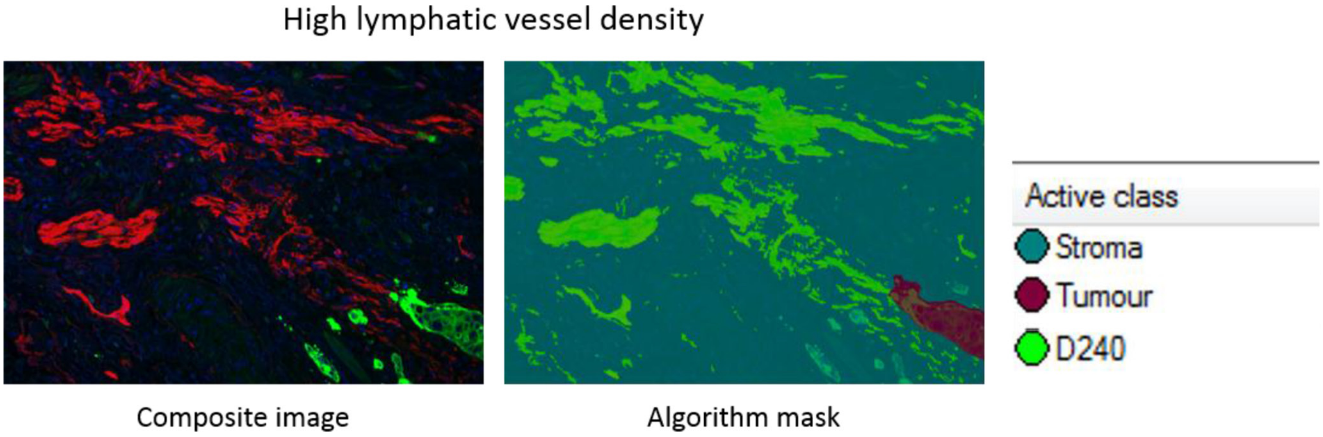

Supplementary Figure S3: Example of high lymphatic vessel density (LVD). Composite image shows an area of high LVD at the invasive front of a CRC tissue section (blue; DAPI, green, panCk & red; lymphatic vessel (D2-40)). The algorithm quantifies the number and area of vessels (green mask) and calculates the lymphatic vessel density.

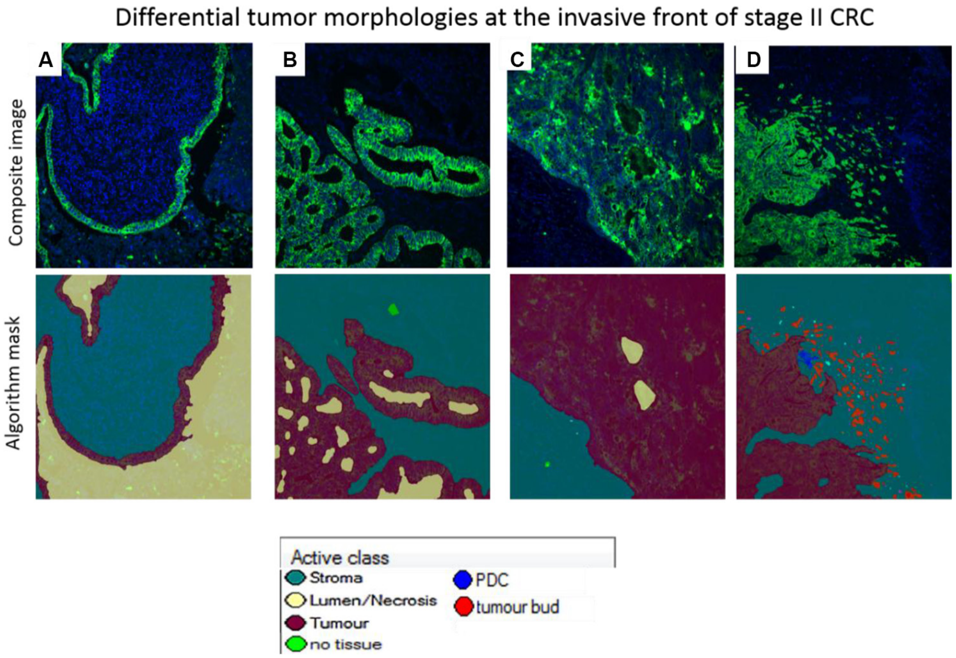

Supplementary Figure S4: Examples of differential tumor morphologies at the invasive front of stage II CRC. Composite images show panCK (epithelial cells) in green and DAPI (nuclei) in blue. The image analysis algorithm segmented and quantified the morphometrics, panCK intensities and tumour, lumen/necrosis and stroma area ratios of the invasive front of CRC. (**A**) Tumor with large area of lumen/necrosis ROI. (**B**) Well differentiated pushing border. (**C**) poorly differentiated pushing border. (**D**) Infiltrative pattern with high tumor budding (red objects in algorithm mask).

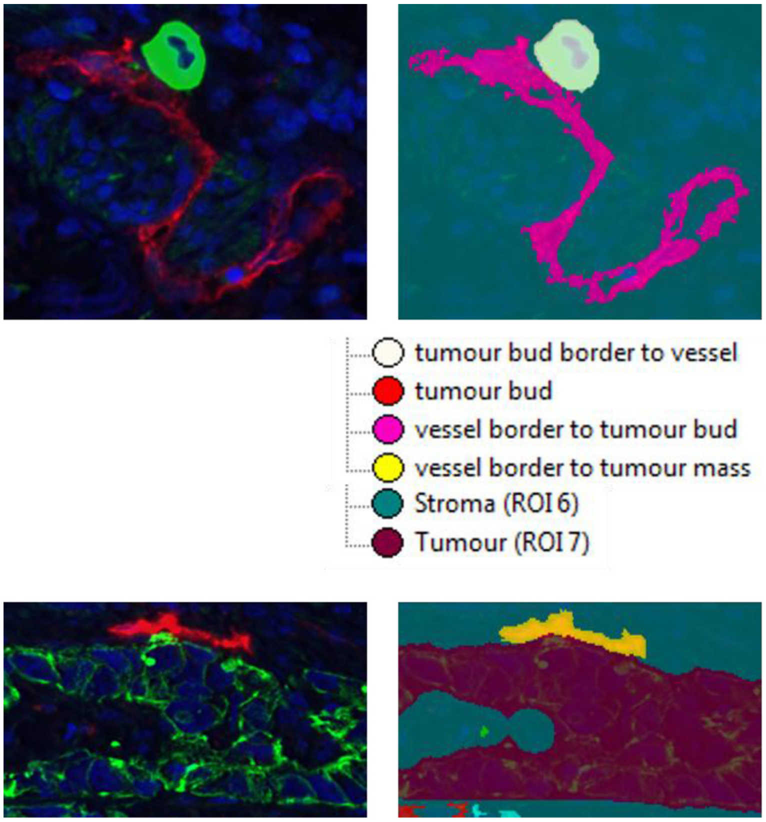

Supplementary Figure S5: Examples of sub-classifications created within the algorithm to capture spatial heterogeneity. (**A**) A tumor bud (white algorithm mask) bordered to a lymphatic vessel (pink algorithm mask) are both counted within the quantification of tumor buds and lymphatic vessel density but are further quantified within these two sub-categories. (**B**) A lymphatic vessel is further sub-classified into the classification 'vessel border to tumor mass' (yellow) as it is bordering a tumor gland (purple).

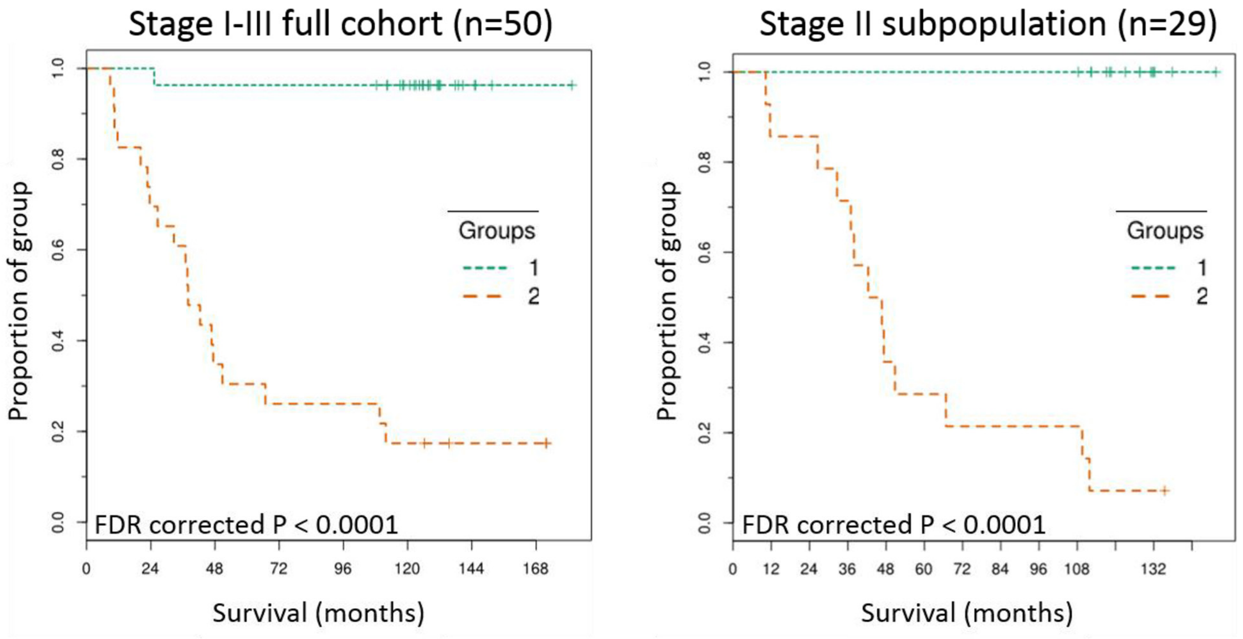

Supplementary Figure S6: Kaplan-Meier curves for Area poorly differentiated clusters (Area PDC). Plots shown for the full training cohort and its stage II subpopulation. Area PDC was highly significant in categorizing patients within the training cohort into high and low risk of disease specific death. *P* values corrected for false discovery rate (FDR). Group1 (green line) patients have an Area PDC below the cut-off of 35,647 µm^2 and group 2 (orange line) have an Area PDC above the cut-off.

Supplementary Table 1: Full list of parameters within the multi-parametric feature set. See Supplementary_Table_S1

SUPPLEMENTARY DOCUMENT 2

Extended method and figures for image analysis algorithm

Algorithm settings listed below

The novel image analysis algorithm was created within the Definiens image analysis software packages Tissue Studio® and Developer XD™ (Definiens AG, Munich). Images were imported into Definiens as .TIFF files using a bespoke importer. Initial image segmentation utilized an image analysis algorithm created in Tissue Studio® as described previously (3). All segmented objects were classified within a hierarchical system where the top level was automatically segmented through machine-based learning using Definiens Composer Technology™ into Regions of Interest (ROIs): ‘tumor’, ‘necrosis/lumen’, ‘no tissue’ and ‘stroma’ (Supplementary Figure 7A, below). PanCK objects which had been classified as ‘tumor’ by the algorithm but were below 5000 µm^2 and contained no lumen were automatically reclassified into the ‘stroma’ ROI (Supplementary Figure 7B, below). These objects, although tumor subpopulations, were non-gland forming and therefore represented tumor buds (≤ 5 associated nuclei) or poorly differentiated clusters (> 5 associated nuclei). By reclassifying these objects into the stroma ROI the algorithm was able to automatically quantify them as separate from neoplastic glands; which were automatically classified as ‘tumor’. Next, the object level of the image analysis hierarchy captured all panCK (marker 1) and D2-40 (marker 2) positive objects in the stroma. The final layer of image analysis identified nuclei through the DAPI channel. Each nucleus was segregated and exclusively belonged within a classified object or region existing in the analysis layers above. The Tissue Studio® analysed workspace was subsequently imported into Developer XD™ for bespoke object classification and optimization. The Developer algorithm was programmed to perform 3 quality control steps to ensure no false reporting due to artefact. Step 1 automatically identified and classified autofluorescence and non-specific staining (such as edge-effect) which may occur when analysing whole tissue sections under

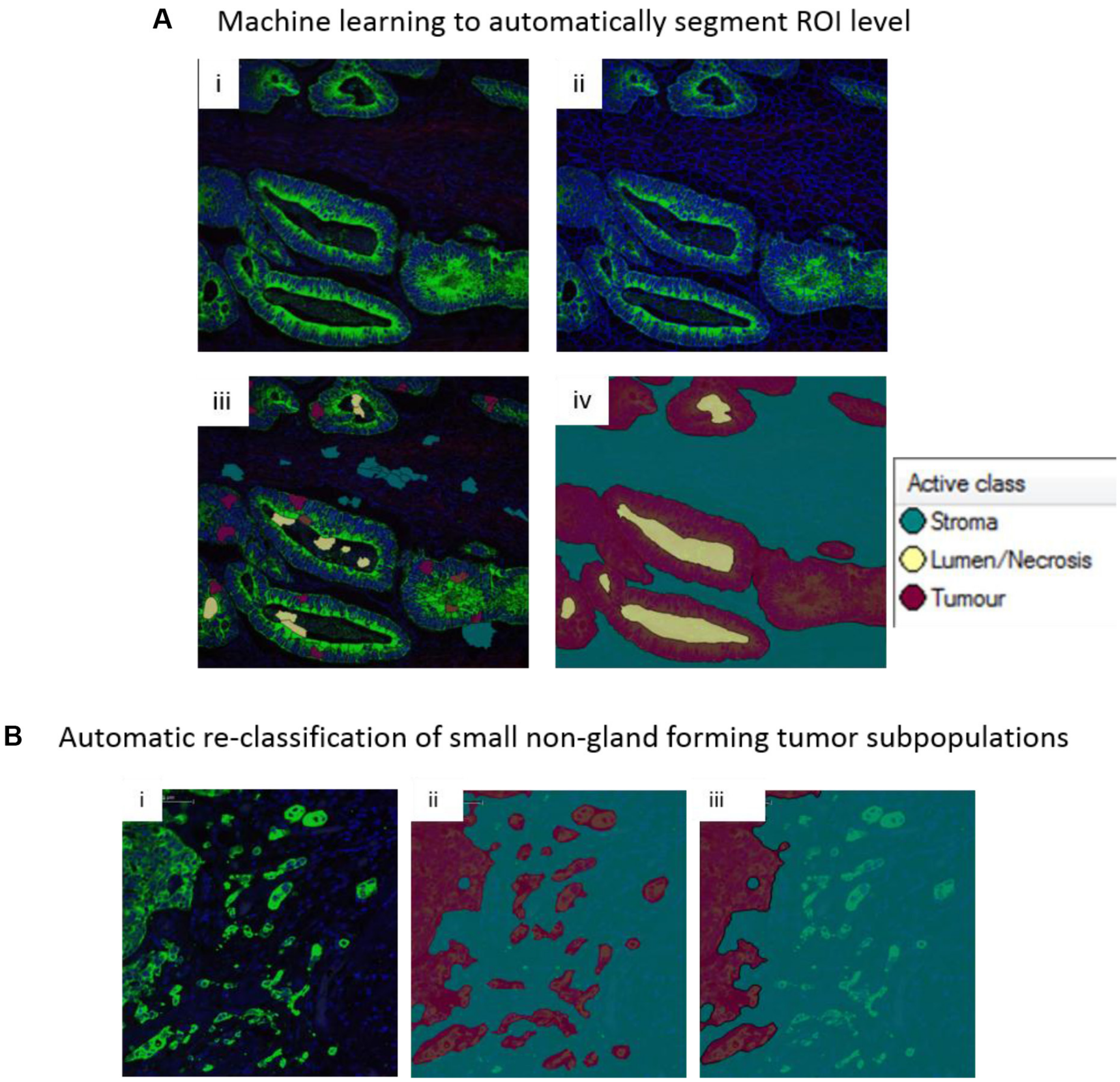

Supplementary Figure S7: Automatic ROI level segmentation and classification. (**A**) The algorithm was trained to automatically segment the ROI level. The composite image (i. blue; DAPI, green; panCK) was automatically segmented by the algorithm (ii. blue segmenting line). iii. The user trained the algorithm to recognize stroma (turquoise), tumor (purple) or lumen/necrosis (yellow) on a subset of images. iv. The algorithm automatically learned and segmented the images at the ROI level within the separate patient cohorts. (**B**) Cytokeratin labelled objects (i) which were under 5000 µm² (e.g. tumor buds and poorly differentiated clusters) and were classified as 'tumor' during machine learning classification (ii) were automatically re-classified to belong to the 'stroma' ROI (iii). This was performed to separate them from tumor glands and allowed them to be quantified separately within the stromal compartment of the ROI image analysis level.

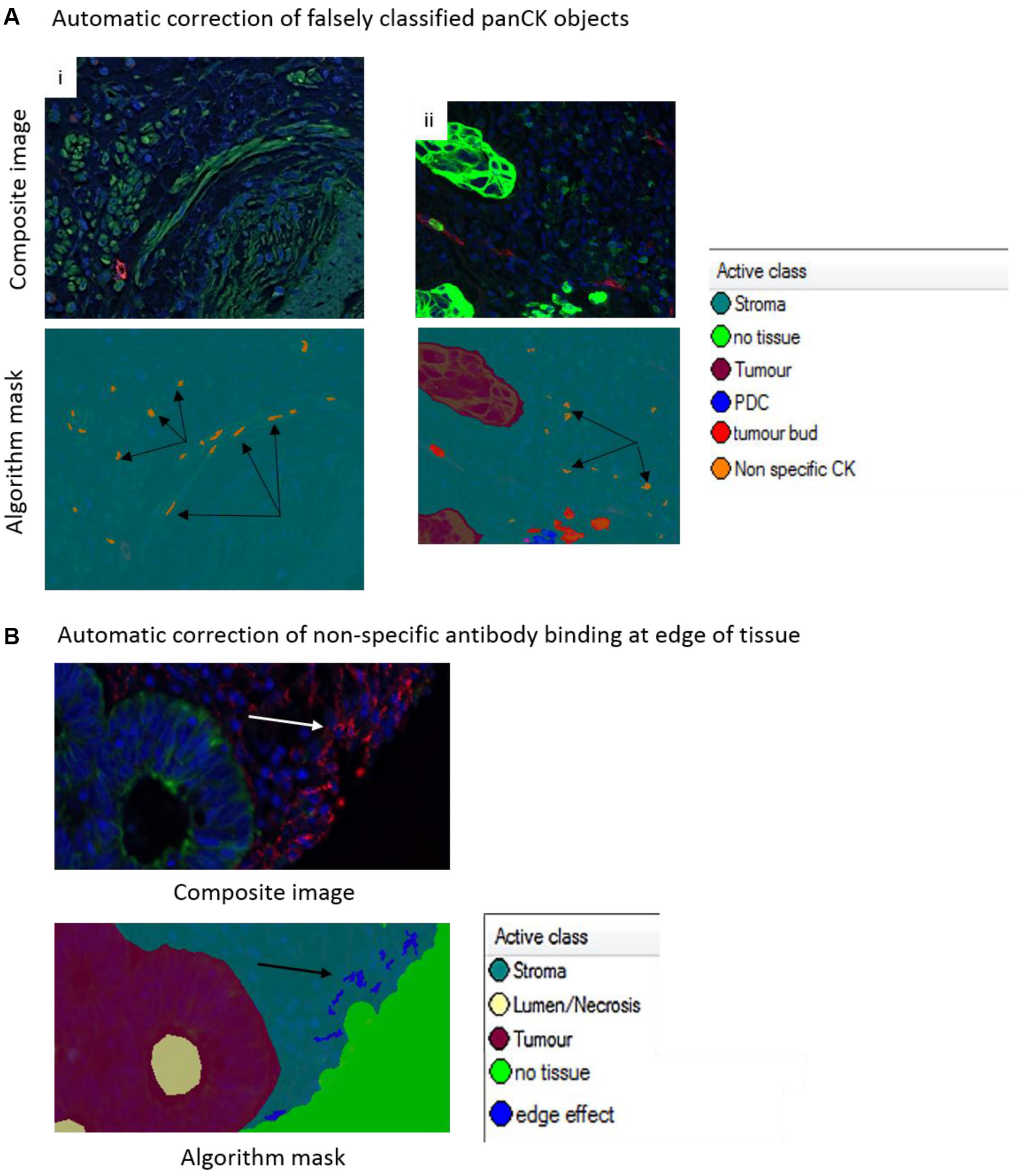

Supplementary Figure S8: Automatic quality control step for autofluorescence and non-specific staining. (**A**)i & ii show areas of tissue autofluorescence captured in the Cy3 channel (panCK – green). The algorithm classified some areas of autofluorescence as panCK positive objects. Automated QC step in algorithm reclassified the autofluorescence segmented objects as 'Non specific CK' (orange algorithm mask), highlighted by black arrows. (**B**) Automatic quality control of edge-effect correction step built into algorithm. Composite image shows non-specific binding of the D2-40 antibody at the edge of a tissue section (red; D2-40, blue; DAPI, green; panCK). To correct for edge-effect any segmented object within 50 µm² of 'no tissue' was automatically re-classified as 'edge-effect' (Dark blue algorithm mask) and not quantified by the algorithm.

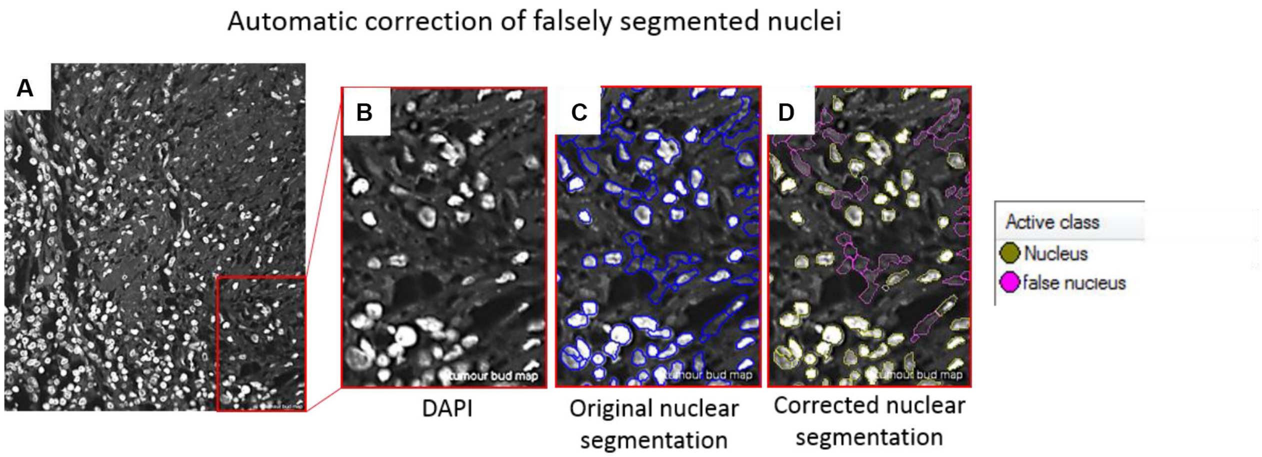

Supplementary Figure S9: Automatic quality control step for nucleus correction built into algorithm. (**A** and **B**). Area of tissue section with high background levels of DAPI channel fluorescence. (**C**) Automated nucleus identification falsely segmented some areas of high DAPI channel autofluorescence as nuclei. (**D**) Automatic step in algorithm re-classified incorrectly segmented nuclei as 'false nucleus' (pink algorithm outline).

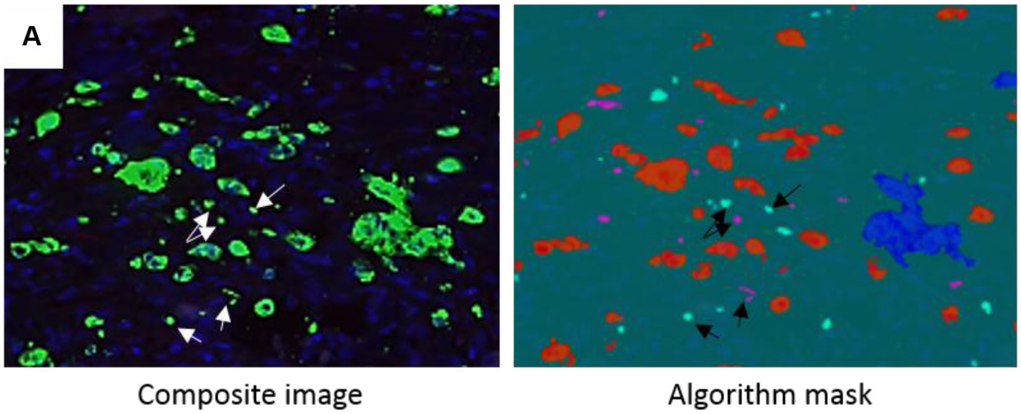

Supplementary Figure 10: Composite image of a high budding area at the invasive front of a CRC tissue section (panCK; green, DAPI; blue) and the corresponding algorithm mask classifying: stroma (turquoise) tumor buds (red), poorly differentiated clusters (dark blue) and arrows highlighting necrotic debris (pink or light blue).

Supplementary Table S2: Training set patient clinicopathology and image analysis data (*n* = 50)

Parameters		univariate HR (95% CI)	*P* value
**Clinical data**	**Category (*n* = patient number)**		
Stage	I (13), II (29), III (8)	3 (1.6–7)	0.001
Gender	M (24), F (26)	2 (0.7–4.3)	0.3
Age	30s (5), 40s (27), 50s (18)	1 (0.9–1)	0.8
pT stage	1 (7), 2 (7), 3 (27), 4 (9)	3 (1.5–6)	0.001
pN stage	0 (40), 1 (8), 2 (1), 3 (1)	2.6 (1.5–4.5)	0.0001
Differentiation	Well (5), Moderate (39), Poor (6)	1 (0.4–3.5)	0.83
Histology	Standard (42), Mucinous (8)	0.5 (0.1–2.1)	0.35
Site	Rectal (17), Right side (17), Left side (16)	0.8 (0.5–1.3)	0.3
**Image analysis data**	**Cut-off (*n* = patient number)**		
TB	287 (13 above, 37 below)	6 (2.4–13.8)	0.0005
LVI	19 16 above, 34 below	13 (4.9–36.7)	< 0.0001
Minimal LVI	16 (17 above, 33 below)	9 (2.5–25)	0.0001
LVD	0.7; D2-40 area % of stroma (19 above, 31 below)	5 (2–13)	0.0001
PDC	35 (12 above, 38 below)	5 (2–11.4)	0.001
Percent Tumor	21 % total tissue (15 below, 35 above)	0.1 (0.05–0.39)	0.0001
Area PDC	35647 μm^2 (23 above, 27 below)	20 (4.6–87.9)	< 0.0001

Clinical patient data was categorized prior to univariate cox-regression. Image analysis data was categorized into above or below cut-off prior to univariate cox-regression.
HR = hazard ratio, CI = confidence interval. TB = tumor budding, LVI = lymphatic vessel invasion, Minimal LVI = minimal lymphatic vessel invasion, LVD = lymphatic vessel density, PDC = poorly differentiated clusters, Percent Tumor = tumor to stroma ratio, Area PDC = area poorly differentiated clusters.

immunofluorescence. (Supplementary Figure 8, below). Step 2 was the classification and deletion of erroneously segmented nuclei (Supplementary Figure 9, below) which may occur from hierarchical image segmentation or from heterogeneous background DAPI intensity across patient samples. Step 3 was the classification of small panCK positive objects (below 50 μm^2) which had no associated nuclei (irrelevant marker) or only debris nuclei associated (debris), both of which are grouped into a classification of 'necrotic debris' (Supplementary Figure 10, below). The Developer algorithm finally calculated and exported the multi-parametric feature set consisting of morpohometric, texture, fluorescence and spatial measurements captured across objects in an unbiased manner.

Definiens tissue studio® algorithm settings

Settings exported as text which were used to program the initial segmentation of the tissue in Tissue studio®

ROI Tissue Segmentation algorithm settings.

Parameter Value
Portal IF Tissue
Action General Settings
Magnification 20
µm/pixel 0.47
Bit depth 12 bit
Layer 1 CK
Layer 2 D240
Layer 3 DAPI
Action Tissue - Background Separation
Select Image Layers CK, D240, DAPI,
Use Autothresholds FALSE
Homogeneity Threshold 0
Brightness Threshold 0
Tissue Min Size (1000 μm^2) 0
Action Composer: Initialization
Magnification 10

Action Composer: Training
Use Layers CK, D240, DAPI,
Action Composer: Reclassify Region
Source Class Tumour
Target Class Stroma
Use Condition TRUE
Condition 1 Area < 5000
Action Initialize Cellular Analysis
Analyze ROI 1 FALSE

Analyze ROI 2	FALSE
Analyze ROI 3	FALSE
Analyze ROI 4	FALSE
Analyze Stroma	TRUE
Analyze ROI 6	FALSE
Analyze ROI 7	FALSE
Analyze ROI 8	FALSE
Magnification	20

Action Marker Area Detection

Marker 1	PanCk
Threshold	35
Marker 2	D240
Threshold	30
Marker	3
Minimum Area (μm²)	5
Export a Screenshot	FALSE

Action Default Export

Export a Screenshot	TRUE
ROI Statistics	TRUE
Cellular Analysis Statistics	TRUE

Definiens devloper XD™ algorithm settings

Settings exported as text which were used to program the DeveloperXD™ algorithm

optimisation of tumour bud and LVI
reset
delete map: on tumour bud map : delete map
delete map: on vessel map : delete map
delete map: on copy of main : delete map
delete map: on LVI colocalisation map: delete map
delete map: on ROI and bud map : delete map
copy map: copy map to 'tumour bud map'
copy map: copy map to 'vessel map'
remove all objects but marker one on tumour bud on tumour bud map
delete image object level: delete 'NucleusLevel'
remove objects: loop: Marker 2 at MarkerLevel: remove objects into unclassified (merge by shape)
assign class: Markers 1 and 2 at MarkerLevel: Marker 1
merge region: Marker 1 at MarkerLevel: merge region
assign class: Marker 2 at MarkerLevel: unclassified
convert image objects: at MarkerLevel: convert image objects -> Connected 2D
merge region: unclassified at MarkerLevel: merge region
assign class: Marker 1 with Existence of super objects Tumour (1) = 1 at MarkerLevel: marker 1 in tumour
assign class: Marker 1 with Existence of super objects necrosis (1) = 1 at MarkerLevel: marker 1 in tumour
assign class: Marker 1 with Existence of super objects no tissue (1) = 1 at MarkerLevel: marker 1 in no tissue
copy image object level: at TissueLevel: copy creating 'CopyofTissueLevel' above
assign class: with Classified as no tissue = 0 at CopyofTissueLevel: _TempClass1
convert image objects: at CopyofTissueLevel: convert image objects -> Connected 2D
merge region: _TempClass1, Tumour at CopyofTissueLevel: merge region
distance map: at CopyofTissueLevel: distance to no tissue(distance map)
delete image object level: delete 'CopyofTissueLevel'
convert to sub-objects: at TissueLevel: convert to sub-objects
assign class: with Existence of sub objects Marker 1 (1) = 1 at TissueLevel: Marker 1
convert image objects: at TissueLevel: convert image objects -> Disconnected (fusion up)
convert image objects: at TissueLevel: convert image objects -> Connected 2D
update variable: Marker 1 at TissueLevel: distance to no tissue = Mean distance map
assign class: Marker 1 with distance to no tissue < = 50 at TissueLevel: edge effect
assign class: Marker 1 with Area < = 55 μm² at TissueLevel: necrotic debris
remove all objects but marker two on vessel map on vessel map
delete image object level: delete 'NucleusLevel'
remove objects: loop: Marker 1 at MarkerLevel: remove objects into unclassified (merge by shape)
assign class: Markers 1 and 2 at MarkerLevel: Marker 2
pixel-based object resizing: loop: on vessel map Marker 2 at MarkerLevel: grow into unclassified where D240 > = 40
find enclosed by class: on vessel map at MarkerLevel: enclosed by Marker 2: Marker 2 +
merge region: on vessel map Marker 2 at MarkerLevel: merge region
assign class: Marker 1 at MarkerLevel: unclassified
convert image objects: at MarkerLevel: convert image objects -> Connected 2D
merge region: unclassified at MarkerLevel: merge region
assign class: Marker 2 with Existence of super objects Tumour (1) = 1 at MarkerLevel: marker 2 in tumour
assign class: Marker 2 with Existence of super objects necrosis (1) = 1 at MarkerLevel: marker

2 in tumour
assign class: Marker 2 with Existence of super objects no tissue (1) = 1 at MarkerLevel: marker 2 in no tissue
copy image object level: at TissueLevel: copy creating ‘CopyofTissueLevel’ above
assign class: with Classified as no tissue = 0 at CopyofTissueLevel: _TempClass2
convert image objects: at CopyofTissueLevel: convert image objects -> Connected 2D
merge region: _TempClass2, Tumour at CopyofTissueLevel: merge region
distance map: at CopyofTissueLevel: distance to no tissue(distance map)
delete image object level: delete ‘CopyofTissueLevel’
convert to sub-objects: at TissueLevel: convert to sub-objects
assign class: with Existence of sub objects Marker 2 (1) = 1 at TissueLevel: Marker 2
convert image objects: at TissueLevel: convert image objects -> Disconnected (fusion up)
convert image objects: at TissueLevel: convert image objects -> Connected 2D
update variable: Marker 2 at TissueLevel: distance to no tissue = Mean distance map
assign class: Marker 2 with distance to no tissue < = 50 at TissueLevel: edge effect
assign class: on vessel map Marker 2 at TissueLevel: D240
evaluate tumour buds
fill holes in buds and evaluate nuclei in buds
pixel-based object resizing: on tumour bud map Marker 1 at TissueLevel: grow into Stroma
find enclosed by class: on tumour bud map at TissueLevel: enclosed by Marker 1: _ TempClass1 +
grow region: on tumour bud map Marker 1 at TissueLevel: <- _TempClass1
merge region: on tumour bud map Marker 1 at TissueLevel: merge region
pixel-based object resizing: on tumour bud map Marker 1 at TissueLevel: shrink using Stroma
convert image objects: at TissueLevel: convert image objects -> Connected 2D
merge region: on tumour bud map Stroma at TissueLevel: merge region
copy image object level: on tumour bud map at TissueLevel: copy creating ‘Nucleus level’ below
synchronize map: on main at NucleusLevel: synchronize map ‘tumour bud map’
assign class: on tumour bud map Nucleus with Area < 16 µm² at Nucleus level: Nucelar debris
assign class: on tumour bud map Nucleus with Standard deviation DAPI < 6 and Mean DAPI < 40 at Nucleus level: false nucleus
assign class: on tumour bud map Marker 1 with Mean CK < = 50 at TissueLevel: Non specific CK
assign class: on tumour bud map Marker 1 with Standard deviation CK < = 10 at TissueLevel: Non specific CK
assign class: on tumour bud map Marker 1 with Number of sub objects Nucleus (1) > 5 and Existence of necrosis (0) = 1 at TissueLevel: PDC
assign class: on tumour bud map Marker 1 with Number of sub objects Nucelar debris (1) > = 1 and Existence of sub objects Nucleus (1) < 1 at TissueLevel: necrotic debris
remove irrelevant marker 1 areas
assign class: on tumour bud map Marker 1 with Existence of sub objects Nucleus (1) < 1 at TissueLevel: necrotic debris
remainder of marker 1 = tumour bud
on tumour bud map
assign class: Marker 1 at TissueLevel: tumour bud
create ROI and Tumour bud map and calculate distance to tumour
copy map: on tumour bud map : copy map to ‘ROI and bud map’
on ROI and bud map
delete image object level: on ROI and bud map : delete ‘Nucleus level’
copy image object level: at TissueLevel: copy creating ‘vessel level’ below
synchronize map: on vessel map at TissueLevel: synchronize map ‘ROI and bud map’
delete layer: delete image layer ‘distance map’
copy image object level: at TissueLevel: copy creating ‘CopyofTissueLevel’ above
assign class: with Classified as Tumour = 0 at CopyofTissueLevel: _TempClass1
convert image objects: at CopyofTissueLevel: convert image objects -> Connected 2D
merge region: _TempClass1 at CopyofTissueLevel: merge region
delete image object level: delete ‘CopyofTissueLevel’
LVI and colocalisation
convert to sub-objects: at TissueLevel: convert to sub-objects
assign class: Stroma with Existence of sub objects D240 (1) = 1 at TissueLevel: D240
assign class: tumour bud with Existence of sub objects D240 (1) = 1 at TissueLevel: minimal LVI
assign class: bud with PDC with Existence of sub objects D240 (1) = 1 at TissueLevel: LVI
assign class: tumour bud with Existence of D240 (0) = 1 at TissueLevel: tumour bud border to marker 2
assign class: bud with PDC with Existence of

D240 (0) = 1 at TissueLevel: PDC border to marker 2
assign class: D240 with Existence of Markers 1 and 2 (0) = 1 at TissueLevel: vessel border to bud invasion
convert image objects: at TissueLevel: convert image objects -> Disconnected (fusion up)
convert image objects: at TissueLevel: convert image objects -> Connected 2D
assign class: D240 with Existence of Tumour (0) = 1 at TissueLevel: vessel border to tumour mass
statistical export of invasive eptihelial bodies
export object statistics: on ROI and bud map D240, necrotic debris, minimal LVI, LVI, tumour bud, tumour bud border to marker 2, vessel border to bud invasion at TissueLevel: export object statistics
export object statistics: on ROI and bud map vessel border to tumour mass at MarkerLevel: export object statistics
export object statistics: on tumour bud map necrotic debris, PDC, tumour bud at TissueLevel: export object statistics
export object statistics: on tumour bud map Nucleus with Existence of super objects tumour bud (1) = 1 at Nucleus level: export object statistics
export object statistics: on tumour bud map Nucleus with Existence of super objects tumour bud (1) = 0 and Existence of super objects Stroma (1) = 1 at Nucleus level: export object statistics
export object statistics: on tumour bud map Nucleus with Existence of super objects Tumour (1) = 1 at Nucleus level: export object statistics
calculate colocalisation stat
update variable: sum of area minimal LVI coloc = 0
compute statistical value: on ROI and bud map minimal LVI at TissueLevel: sum of area minimal LVI = sum(Area)
update variable: sum of area LVI = 0
compute statistical value: on ROI and bud map PDC invasion at TissueLevel: sum of area LVI = sum(Area)
update variable: sum of area of vessel border to bud invasion = 0
compute statistical value: on ROI and bud map vessel border to bud invasion at TissueLevel: sum of area of vessel border to bud invasion = sum(Area)
update variable: number of bordering bud and vessel = 0
compute statistical value: on ROI and bud map tumour bud border to marker 2 at TissueLevel: number of bordering bud and vessel = number
update variable: number of bordering tumour and vessel = 0
compute statistical value: on ROI and bud map PDC border to marker 2 at TissueLevel: number of bordering PDC and vessel = number
update variable: number of vessel border to bud invasion = 0
compute statistical value: on ROI and bud map vessel border to bud invasion at TissueLevel: number of vessel border to bud invasion = number
update variable: number of minimal LVI = 0
compute statistical value: on ROI and bud map Markers 1 and 2 at TissueLevel: number of minimal LVI = number
update variable: number of LVI = 0
compute statistical value: on ROI and bud map LVI at TissueLevel: number of LVI = number
calculate tumour bud stat
update variable: number of buds = 0
compute statistical value: on tumour bud map tumour bud at TissueLevel: number of buds = number
update variable: no. of PDC = 0
compute statistical value: on tumour bud map PDC at TissueLevel: no. of PDC = number
update variable: bud with < = 2 nuc = 0
compute statistical value: on tumour bud map tumour bud with Number of sub objects Nucleus (1) < = 2 at TissueLevel: bud with < = 2 nuc = number
update variable: bud with > = 3 nuc = 0
compute statistical value: on tumour bud map tumour bud with Number of sub objects Nucleus (1) > = 3 at TissueLevel: bud with > = 3 nuc = number
update variable: sum area of buds = 0
compute statistical value: on tumour bud map tumour bud at TissueLevel: sum area of buds = sum(Area)
update variable: on tumour bud map tumour bud at TissueLevel: Individual bud area = Area
calculate vessel stat
update variable: number of vessels = 0
compute statistical value: on vessel map D240 with Existence of Stroma (0) = 1 at TissueLevel: number of vessels = number
update variable: sum area of vessels = 0
compute statistical value: on vessel map D240 at TissueLevel: sum area of vessels = sum(Area)
update variable: on vessel map D240 at TissueLevel: Individual vessel area = Area
calculate mean nuclear stats in stroma
update variable: nuc in stroma_meanD240 = 0

compute statistical value: on tumour bud map Nucleus with Existence of super objects Stroma (1) = 1 at Nucleus level: nuc in stroma_meanD240 = mean(Mean D240)
update variable: nuc in stroma_meanCK = 0
compute statistical value: on tumour bud map Nucleus with Existence of super objects Stroma (1) = 1 at Nucleus level: nuc in stroma_meanCK = mean(Mean CK)
update variable: nuc in stroma_meanDAPI = 0
compute statistical value: on tumour bud map Nucleus with Existence of super objects Stroma (1) = 1 at Nucleus level: nuc in stroma_meanDAPI = mean(Mean DAPI)
update variable: nuc in stroma_mean_stdev_CK = 0
compute statistical value: on tumour bud map Nucleus with Existence of super objects Stroma (1) = 1 at Nucleus level: nuc in stroma_mean_stdev_CK = mean(Standard deviation CK)
update variable: nuc in stroma_mean_stdev_D240 = 0
compute statistical value: on tumour bud map Nucleus with Existence of super objects Stroma (1) = 1 at Nucleus level: nuc in stroma_mean_stdev_D240 = mean(Standard deviation D240)
update variable: nuc in stroma_mean_stdev_DAPI = 0
compute statistical value: on tumour bud map Nucleus with Existence of super objects Stroma (1) = 1 at Nucleus level: nuc in stroma_mean_stdev_DAPI = mean(Standard deviation DAPI)
update variable: nuc in stroma_mean_Ratio_CK = 0
compute statistical value: on tumour bud map Nucleus with Existence of super objects Stroma (1) = 1 at Nucleus level: nuc in stroma_mean_Ratio_CK = mean(Ratio CK)
update variable: nuc in stroma_mean_Ratio_D240 = 0
compute statistical value: on tumour bud map Nucleus with Existence of super objects Stroma (1) = 1 at Nucleus level: nuc in stroma_mean_Ratio_D240 = mean(Ratio D240)
update variable: nuc in stroma_mean_Ratio_DAPI = 0
compute statistical value: on tumour bud map Nucleus with Existence of super objects Stroma (1) = 1 at Nucleus level: nuc in stroma_mean_Ratio_DAPI = mean(Ratio DAPI)
update variable: nuc in stroma_mean_Area = 0
compute statistical value: on tumour bud map Nucleus with Existence of super objects Stroma (1) = 1 at Nucleus level: nuc in stroma_mean_Area = mean(Area)
update variable: nuc in stroma_mean_borderlength = 0
compute statistical value: on tumour bud map Nucleus with Existence of super objects Stroma (1) = 1 at Nucleus level: nuc in stroma_mean_borderlength = mean(Border length)
update variable: nuc in stroma_mean_length = 0
compute statistical value: on tumour bud map Nucleus with Existence of super objects Stroma (1) = 1 at Nucleus level: nuc in stroma_mean_length = mean(Length)
update variable: nuc in stroma_asymmetry = 0
compute statistical value: on tumour bud map Nucleus with Existence of super objects Stroma (1) = 1 at Nucleus level: nuc in stroma_asymmetry = mean(Asymmetry)
update variable: nuc in stroma_border index = 0
compute statistical value: on tumour bud map Nucleus with Existence of super objects Stroma (1) = 1 at Nucleus level: nuc in stroma_border index = mean(Border index)
update variable: nuc in stroma_mean_lengthwidth = 0
compute statistical value: on tumour bud map Nucleus with Existence of super objects Stroma (1) = 1 at Nucleus level: nuc in stroma_mean_lengthwidth = mean(Length\Width)
update variable: nuc in stroma_mean_perimeter = 0
compute statistical value: on tumour bud map Nucleus with Existence of super objects Stroma (1) = 1 at Nucleus level: nuc in stroma_mean_perimeter = mean(Perimeter)
update variable: nuc in stroma_mean_width = 0
compute statistical value: on tumour bud map Nucleus with Existence of super objects Stroma (1) = 1 at Nucleus level: nuc in stroma_mean_width = mean(Width)
update variable: nuc in stroma_mean_circularity = 0
compute statistical value: on tumour bud map Nucleus with Existence of super objects Stroma (1) = 1 at Nucleus level: nuc in stroma_mean_circularity = mean(Circularity)
update variable: nuc in stroma_mean_compactness = 0
compute statistical value: on tumour bud map Nucleus with Existence of super objects Stroma (1) = 1 at Nucleus level: nuc in stroma_mean_compactness = mean(Compactness)
update variable: nuc in stroma_mean_density = 0
compute statistical value: on tumour bud map Nucleus with Existence of super objects Stroma (1) = 1 at Nucleus level: nuc in stroma_mean_density = mean(Density)
update variable: nuc in stroma_mean_

Ellipticity = 0
compute statistical value: on tumour bud map Nucleus with Existence of super objects Stroma (1) = 1 at Nucleus level: nuc in stroma_mean_Ellipticity = mean(Ellipticity)
update variable: nuc in stroma_mean_Roundness = 0
compute statistical value: on tumour bud map Nucleus with Existence of super objects Stroma (1) = 1 at Nucleus level: nuc in stroma_mean_Roundness = mean(Roundness)
update variable: nuc in stroma_mean_Shape index = 0
compute statistical value: on tumour bud map Nucleus with Existence of super objects Stroma (1) = 1 at Nucleus level: nuc in stroma_mean_Shape index = mean(Shape index)
calculate mean nuclear stats in bud
update variable: nuc in bud_meanD240 = 0
compute statistical value: on tumour bud map Nucleus with Existence of super objects tumour bud (1) = 1 at Nucleus level: nuc in bud_meanD240 = mean(Mean D240)
update variable: nuc in bud_meanCK = 0
compute statistical value: on tumour bud map Nucleus with Existence of super objects tumour bud (1) = 1 at Nucleus level: nuc in bud_meanCK = mean(Mean CK)
update variable: nuc in bud_meanDAPI = 0
compute statistical value: on tumour bud map Nucleus with Existence of super objects tumour bud (1) = 1 at Nucleus level: nuc in bud_meanDAPI = mean(Mean DAPI)
update variable: nuc in bud_mean_stdev_CK = 0
compute statistical value: on tumour bud map Nucleus with Existence of super objects tumour bud (1) = 1 at Nucleus level: nuc in bud_mean_stdev_CK = mean(Standard deviation CK)
update variable: nuc in bud_mean_stdev_D240 = 0
compute statistical value: on tumour bud map Nucleus with Existence of super objects tumour bud (1) = 1 at Nucleus level: nuc in bud_mean_stdev_D240 = mean(Standard deviation D240)
update variable: nuc in bud_mean_stdev_DAPI = 0
compute statistical value: on tumour bud map Nucleus with Existence of super objects tumour bud (1) = 1 at Nucleus level: nuc in bud_mean_stdev_DAPI = mean(Standard deviation DAPI)
update variable: nuc in bud_mean_Ratio_CK = 0
compute statistical value: on tumour bud map Nucleus with Existence of super objects tumour bud (1) = 1 at Nucleus level: nuc in bud_mean_Ratio_CK = mean(Ratio CK)
update variable: nuc in bud_mean_Ratio_D240 = 0
compute statistical value: on tumour bud map Nucleus with Existence of super objects tumour bud (1) = 1 at Nucleus level: nuc in bud_mean_Ratio_D240 = mean(Ratio D240)
update variable: nuc in bud_mean_Ratio_DAPI = 0
compute statistical value: on tumour bud map Nucleus with Existence of super objects tumour bud (1) = 1 at Nucleus level: nuc in bud_mean_Ratio_DAPI = mean(Ratio DAPI)
update variable: nuc in bud_mean_Area = 0
compute statistical value: on tumour bud map Nucleus with Existence of super objects tumour bud (1) = 1 at Nucleus level: nuc in bud_mean_Area = mean(Area)
update variable: nuc in bud_mean_borderlength = 0
compute statistical value: on tumour bud map Nucleus with Existence of super objects tumour bud (1) = 1 at Nucleus level: nuc in bud_mean_borderlength = mean(Border length)
update variable: nuc in bud_mean_length = 0
compute statistical value: on tumour bud map Nucleus with Existence of super objects tumour bud (1) = 1 at Nucleus level: nuc in bud_mean_length = mean(Length)
update variable: nuc in bud_asymmetry = 0
compute statistical value: on tumour bud map Nucleus with Existence of super objects tumour bud (1) = 1 at Nucleus level: nuc in bud_asymmetry = mean(Asymmetry)
update variable: nuc in bud_border index = 0
compute statistical value: on tumour bud map Nucleus with Existence of super objects tumour bud (1) = 1 at Nucleus level: nuc in bud_border index = mean(Border index)
update variable: nuc in bud_mean_lengthwidth = 0
compute statistical value: on tumour bud map Nucleus with Existence of super objects tumour bud (1) = 1 at Nucleus level: nuc in bud_mean_lengthwidth = mean(Length\Width)
update variable: nuc in bud_mean_perimeter = 0
compute statistical value: on tumour bud map Nucleus with Existence of super objects tumour bud (1) = 1 at Nucleus level: nuc in bud_mean_perimeter = mean(Perimeter)
update variable: nuc in bud_mean_width = 0
compute statistical value: on tumour bud map Nucleus with Existence of super objects tumour bud (1) = 1 at Nucleus level: nuc in bud_mean_width = mean(Width)
update variable: nuc in bud_mean_circularity = 0
compute statistical value: on tumour bud map Nucleus with Existence of super objects tumour

bud (1) = 1 at Nucleus level: nuc in bud_mean_circularity = mean(Circularity)
update variable: nuc in bud_mean_compactness = 0
compute statistical value: on tumour bud map Nucleus with Existence of super objects tumour bud (1) = 1 at Nucleus level: nuc in bud_mean_compactness = mean(Compactness)
update variable: nuc in bud_mean_density = 0
compute statistical value: on tumour bud map Nucleus with Existence of super objects tumour bud (1) = 1 at Nucleus level: nuc in bud_mean_density = mean(Density)
update variable: nuc in bud_mean_Ellipticity = 0
compute statistical value: on tumour bud map Nucleus with Existence of super objects tumour bud (1) = 1 at Nucleus level: nuc in bud_mean_Ellipticity = mean(Ellipticity)
update variable: nuc in bud_mean_Roundness = 0
compute statistical value: on tumour bud map Nucleus with Existence of super objects tumour bud (1) = 1 at Nucleus level: nuc in bud_mean_Roundness = mean(Roundness)
update variable: nuc in bud_mean_Shape index = 0
compute statistical value: on tumour bud map Nucleus with Existence of super objects tumour bud (1) = 1 at Nucleus level: nuc in bud_mean_Shape index = mean(Shape index)
calculate mean nuclear stats in tumour
update variable: nuc in tumour_meanD240 = 0
compute statistical value: on tumour bud map Nucleus with Existence of super objects Tumour (1) = 1 at Nucleus level: nuc in tumour_meanD240 = mean(Mean D240)
update variable: nuc in tumour_meanCK = 0
compute statistical value: on tumour bud map Nucleus with Existence of super objects Tumour (1) = 1 at Nucleus level: nuc in tumour_meanCK = mean(Mean CK)
update variable: nuc in tumour_meanDAPI = 0
compute statistical value: on tumour bud map Nucleus with Existence of super objects Tumour (1) = 1 at Nucleus level: nuc in tumour_meanDAPI = mean(Mean DAPI)
update variable: nuc in tumour_mean_stdev_CK = 0
compute statistical value: on tumour bud map Nucleus with Existence of super objects Tumour (1) = 1 at Nucleus level: nuc in tumour_mean_stdev_CK = mean(Standard deviation CK)
update variable: nuc in tumour_mean_stdev_D240 = 0
compute statistical value: on tumour bud map Nucleus with Existence of super objects Tumour (1) = 1 at Nucleus level: nuc in tumour_mean_stdev_D240 = mean(Standard deviation D240)
update variable: nuc in tumour_mean_stdev_DAPI = 0
compute statistical value: on tumour bud map Nucleus with Existence of super objects Tumour (1) = 1 at Nucleus level: nuc in tumour_mean_stdev_DAPI = mean(Standard deviation DAPI)
update variable: nuc in tumour_mean_Ratio_CK = 0
compute statistical value: on tumour bud map Nucleus with Existence of super objects Tumour (1) = 1 at Nucleus level: nuc in tumour_mean_Ratio_CK = mean(Ratio CK)
update variable: nuc in tumour_mean_Ratio_D240 = 0
compute statistical value: on tumour bud map Nucleus with Existence of super objects Tumour (1) = 1 at Nucleus level: nuc in tumour_mean_Ratio_D240 = mean(Ratio D240)
update variable: nuc in tumour_mean_Ratio_DAPI = 0
compute statistical value: on tumour bud map Nucleus with Existence of super objects Tumour (1) = 1 at Nucleus level: nuc in tumour_mean_Ratio_DAPI = mean(Ratio DAPI)
update variable: nuc in tumour_mean_Area = 0
compute statistical value: on tumour bud map Nucleus with Existence of super objects Tumour (1) = 1 at Nucleus level: nuc in tumour_mean_Area = mean(Area)
update variable: nuc in tumour_mean_borderlength = 0
compute statistical value: on tumour bud map Nucleus with Existence of super objects Tumour (1) = 1 at Nucleus level: nuc in tumour_mean_borderlength = mean(Border length)
update variable: nuc in tumour_mean_length = 0
compute statistical value: on tumour bud map Nucleus with Existence of super objects Tumour (1) = 1 at Nucleus level: nuc in tumour_mean_length = mean(Length)
update variable: nuc in tumour_asymmetry = 0
compute statistical value: on tumour bud map Nucleus with Existence of super objects Tumour (1) = 1 at Nucleus level: nuc in tumour_asymmetry = mean(Asymmetry)
update variable: nuc in tumour_border index = 0
compute statistical value: on tumour bud map Nucleus with Existence of super objects Tumour (1) = 1 at Nucleus level: nuc in tumour_border index = mean(Border index)
update variable: nuc in tumour_mean_lengthwidth = 0
compute statistical value: on tumour bud map

Nucleus with Existence of super objects Tumour (1) = 1 at Nucleus level: nuc in tumour_mean_lengthwidth = mean(Length\Width)
update variable: nuc in tumour_mean_perimeter = 0
compute statistical value: on tumour bud map Nucleus with Existence of super objects Tumour (1) = 1 at Nucleus level: nuc in tumour_mean_perimeter = mean(Perimeter)
update variable: nuc in tumour_mean_width = 0
compute statistical value: on tumour bud map Nucleus with Existence of super objects Tumour (1) = 1 at Nucleus level: nuc in tumour_mean_width = mean(Width)
update variable: nuc in tumour_mean_circularity = 0
compute statistical value: on tumour bud map Nucleus with Existence of super objects Tumour (1) = 1 at Nucleus level: nuc in tumour_mean_circularity = mean(Circularity)
update variable: nuc in tumour_mean_compactness = 0
compute statistical value: on tumour bud map Nucleus with Existence of super objects Tumour (1) = 1 at Nucleus level: nuc in tumour_mean_compactness = mean(Compactness)
update variable: nuc in tumour_mean_density = 0
compute statistical value: on tumour bud map Nucleus with Existence of super objects Tumour (1) = 1 at Nucleus level: nuc in tumour_mean_density = mean(Density)
update variable: nuc in tumour_mean_Ellipticity = 0
compute statistical value: on tumour bud map Nucleus with Existence of super objects Tumour (1) = 1 at Nucleus level: nuc in tumour_mean_Ellipticity = mean(Ellipticity)
update variable: nuc in tumour_mean_Roundness = 0
compute statistical value: on tumour bud map Nucleus with Existence of super objects Tumour (1) = 1 at Nucleus level: nuc in tumour_mean_Roundness = mean(Roundness)
update variable: nuc in tumour_mean_Shape index = 0
compute statistical value: on tumour bud map Nucleus with Existence of super objects Tumour (1) = 1 at Nucleus level: nuc in tumour_mean_Shape index = mean(Shape index)
export LVI and bud stats
export project statistics: on ROI and bud map : export project statistics
export project statistics: on tumour bud map : export project statistics
export object statistics: on ROI and bud map Minimal LVI, LVI, PDC border to marker 2, tumour bud border to marker 2, vessel border to minimal LVI at TissueLevel: export object statistics
export object statistics: on tumour bud map unclassified at Nucleus level: export object statistics
export object statistics: on tumour bud map Stroma at Nucleus level: export object statistics
calculate LVD and export vessel stats
export project statistics: on vessel map : export project statistics
export object statistics: on vessel map D240 at TissueLevel: export object statistics
