# Supplementary material for: Novel histopathologic feature identified through image analysis augments stage II colorectal cancer clinical reporting
Source: Oncotarget. 2016 Jun 15;7(28):44381–94. doi: 10.18632/oncotarget.10053 (PMC5190104; doi:10.18632/oncotarget.10053)
Supplement: Supplementary file 2 [file oncotarget-07-44381-s002.docx]

Supplementary Table 1: Full list of parameters within the multi-parametric feature set

| **Variable** | **Collation method** | **Gini Score** |
| --- | --- | --- |
| **AREA_OF_PDC_PXL** | Sum | 3.1 |
| **AREA_LVI** | Sum | 2.93 |
| **AREA_MINIMAL_LVI** | Sum | 2.58 |
| **No_OF_VESSEL_BORDER_TO_MINIMAL_LVI** | Sum | 2.56 |
| **No_OF_PDC** | Sum | 2.43 |
| **No_OF_LVI** | Sum | 2.21 |
| **No_OF_MINIMAL_LVI** | Sum | 2.14 |
| **No_OF_BORDERING_BUD_AND_VESSEL** | Sum | 2.05 |
| **No_OF_BORDERING_TUMOR_GLAND_AND_VESSEL** | Sum | 1.95 |
| **BUD_WITH_≥3_NUC** | Sum | 1.94 |
| **TUMOR_RELATIVE_AREA** | Mean | 1.92 |
| **BUD_WITH_≤2_NUC** | Sum | 1.92 |
| **AREA_OF_VESSEL_BORDER_TO_MINIMAL_LVI_PXL** | Sum | 1.9 |
| **STROMA_RELATIVE_AREA** | Mean | 1.86 |
| **No_OF_BUDS** | Sum | 1.81 |
| **AREA_OF_BUDS** | Sum | 1.8 |
| **NUC_IN_STROMA_MEAN_DENSITY** | Mean | 1.78 |
| **No_OF_NECROTIC_DEBRIS_MARKER** | Sum | 1.64 |
| **AREA_OF_VESSEL_BORDER_TO_LVI** | Sum | 1.64 |
| **NECROSIS_RELATIVE_AREA** | Mean | 1.62 |
| **TUMOR_BUD_AREA_PERCENTAGE_OF_STROMA** | Mean | 1.57 |
| **AREA_OF_DEBRIS_MARKER_PXL** | Sum | 1.49 |
| **NUC_IN_BUD_MEAN_LENGTH** | Mean | 1.44 |
| **No_OF_VESSELS** | Sum | 1.43 |
| **NUC_IN_STROMA_MEAN_STDEV_CK** | Mean | 1.42 |
| **AREA_OF_VESSELS** | Sum | 1.39 |
| **AREA_OF_NECROTIC_DEBRIS_MARKER_PXL** | Sum | 1.36 |
| **No_OF_DEBRIS_MARKER** | Sum | 1.32 |
| **NUC_IN_BUD_MEAN_AREA** | Mean | 1.26 |
| **D240_AREA_PERCENTAGE_OF_STROMA** | Mean | 1.25 |
| **AVERAGE_CK_INTENSITY_STROMA** | Mean | 1.23 |
| **NUC_IN_STROMA_ASYMMETRY** | Mean | 1.12 |
| **NUC_IN_STROMA_MEAN_LENGTHWIDTH** | Mean | 1.11 |
| **NUC_IN_TUMOR_MEAN_STDEV_CK** | Mean | 1.08 |
| **NUC_IN_TUMOR_MEAN_RATIO_D240** | Mean | 1.04 |
| **LAYER_MEAN_OF_CK_PDC** | Mean | 1.04 |
| **NUC_IN_BUD_MEAN_DAPI** | Mean | 0.99 |
| **NUC_IN_BUD_MEAN_WIDTH** | Mean | 0.95 |
| **MEAN_PERIMETER_TUMOR** | Mean | 0.91 |
| **NUC_IN_BUD_MEAN_BORDERLENGTH** | Mean | 0.91 |
| **REL_BORDER_TO_STROMA_TUMOR** | Mean | 0.83 |
| **No_OF_VESSEL_BORDER_TO_TUMOR_GLAND** | Sum | 0.8 |
| **BORDER_TO_STROMA_TUMOR** | Mean | 0.8 |
| **BORDER_INDEX_TUMOR** | Mean | 0.8 |
| **MEAN_CK_TUMOR** | Mean | 0.74 |
| **NUC_IN_STROMA_MEAN_COMPACTNESS** | Mean | 0.74 |
| **AREA_OF_VESSEL_BORDER_TO_TUMOR_MASS_PXL** | Sum | 0.72 |
| **NUC_IN_STROMA_MEAN_SHAPE_INDEX** | Mean | 0.71 |
| **WIDTH_TUMOR** | Mean | 0.69 |
| **NUC_IN_STROMA_MEANCK** | Mean | 0.69 |
| **NUC_IN_BUD_MEAN_STDEV_DAPI** | Mean | 0.69 |
| **AREA_TUMOR** | Mean | 0.67 |
| **NUC_IN_TUMOR_MEAN_RATIO_DAPI** | Mean | 0.67 |
| **NUC_IN_STROMA_MEAN_CIRCULARITY** | Mean | 0.63 |
| **NUC_IN_STROMA_MEAN_RATIO_CK** | Mean | 0.63 |
| **NUC_IN_BUD_MEAN_STDEV_D240** | Mean | 0.62 |
| **NUC_IN_STROMA_MEAN_ROUNDNESS** | Mean | 0.58 |
| **NUC_IN_BUD_MEAN_DENSITY** | Mean | 0.56 |
| **NUC_IN_STROMA_MEAN_D240** | Mean | 0.54 |
| **AVERAGE_CK_INTENSITY_TUMOR_AREA** | Mean | 0.54 |
| **NUC_IN_TUMOR_MEAN_STDEV_DAPI** | Mean | 0.53 |
| **NUC_IN_TUMOR_MEAN_RATIO_CK** | Mean | 0.52 |
| **NUC_IN_STROMA_MEAN_ELLIPTICITY** | Mean | 0.49 |
| **NUC_IN_BUD_MEANCK** | Mean | 0.48 |
| **ASYMMETRY__TUMOR** | Mean | 0.48 |
| **NUC_IN_STROMA_MEAN_RATIO_D240** | Mean | 0.47 |
| **NUC_IN_STROMA_MEAN_STDEV_DAPI** | Mean | 0.44 |
| **NUC_IN_TUMOR_MEAN_COMPACTNESS** | Mean | 0.43 |
| **NUC_IN_STROMA_MEAN_STDEV_D240** | Mean | 0.43 |
| **NUC_IN_TUMOR_MEANCK** | Mean | 0.43 |
| **NUC_IN_BUD_MEAN_RATIO_D240** | Mean | 0.43 |
| **NUC_IN_BUD_MEAN_RATIO_CK** | Mean | 0.42 |
| **MEAN_DAPI_TUMOR** | Mean | 0.4 |
| **NUC_IN_BUD_MEAND240** | Mean | 0.4 |
| **NUC_IN_STROMA_MEAN_RATIO_DAPI** | Mean | 0.4 |
| **LAYER_MEAN_OF_CK_TUMOR_BUD** | Mean | 0.39 |
| **NUC_IN_TUMOR_MEAN_LENGTHWIDTH** | Mean | 0.39 |
| **NUC_IN_STROMA_BORDER_INDEX** | Mean | 0.38 |
| **NUC_IN_TUMOR_MEANDAPI** | Mean | 0.38 |
| **NUC_IN_BUD_MEAN_SHAPE_INDEX** | Mean | 0.37 |
| **NUC_IN_STROMA_MEANDAPI** | Mean | 0.37 |
| **ELLIPTIC_FIT__TUMOR** | Mean | 0.37 |
| **LENGTH_WIDTH__TUMOR** | Mean | 0.36 |
| **NUC_IN_TUMOR_MEAN_STDEV_D240** | Mean | 0.35 |
| **NUC_IN_BUD_MEAN_RATIO_DAPI** | Mean | 0.35 |
| **NUC_IN_TUMOR_ASYMMETRY** | Mean | 0.34 |
| **NUC_IN_STROMA_MEAN_WIDTH** | Mean | 0.33 |
| **LENGTH_TUMOR** | Mean | 0.31 |
| **MEAN_D240_TUMOR** | Mean | 0.31 |
| **NUC_IN_BUD_MEAN_COMPACTNESS** | Mean | 0.3 |
| **NUC_IN_BUD_MEAN_STDEV_CK** | Mean | 0.3 |
| **NUC_IN_TUMOR_MEAN_CIRCULARITY** | Mean | 0.3 |
| **LAYER_MEAN_OF_CK_DEBRIS_MARKER_NUCLEUS** | Mean | 0.3 |
| **NUC_IN_TUMOR_MEAN_SHAPE_INDEX** | Mean | 0.29 |
| **AVERAGE_DAPI_INTENSITY_TUMOR_AREA** | Mean | 0.28 |
| **NUC_IN_BUD_MEAN_ELLIPTICITY** | Mean | 0.28 |
| **MEAN_COMPACTNESS_TUMOR** | Mean | 0.27 |
| **MEAN_OF_D240_VESSEL** | Mean | 0.27 |
| **DAPI_INTENSITY_STROMA** | Mean | 0.26 |
| **ROUNDNESS_TUMOR** | Mean | 0.26 |
| **NUC_IN_TUMOR_MEAN_DENSITY** | Mean | 0.26 |
| **NUC_IN_TUMOR_MEAN_D240** | Mean | 0.26 |
| **NUC_IN_BUD_MEAN_CIRCULARITY** | Mean | 0.25 |
| **NUC_IN_BUD_MEAN_LENGTHWIDTH** | Mean | 0.25 |
| **NUC_IN_TUMOR_MEAN_LENGTH** | Mean | 0.25 |
| **MEAN_ELLIPTICITY_TUMOR** | Mean | 0.25 |
| **AREA_OF_TUMOR_INVADING_VESSEL** | Sum | 0.25 |
| **NUC_IN_BUD_ASYMMETRY** | Mean | 0.24 |
| **MEAN_RECTANGULAR_FIT_TUMOR** | Mean | 0.24 |
| **NUC_IN_STROMA_MEAN_AREA** | Mean | 0.24 |
| **NUMBER_OF_TUMOR_INVADING_VESSEL** | Sum | 0.24 |
| **NUC_IN_TUMOR_BORDER_INDEX** | Mean | 0.24 |
| **NUC_IN_BUD_BORDER_INDEX** | Mean | 0.23 |
| **MEAN_OF_CK_NECROTIC_DEBRIS_MARKER** | Mean | 0.23 |
| **NUC_IN_BUD_MEAN_ROUNDNESS** | Mean | 0.23 |
| **NUC_IN_STROMA_MEAN_LENGTH** | Mean | 0.23 |
| **NUC_IN_TUMOR_MEAN_WIDTH** | Mean | 0.22 |
| **NUC_IN_TUMOR_MEAN_ELLIPTICITY** | Mean | 0.22 |
| **CIRCULARITY_TUMOR** | Mean | 0.22 |
| **NUC_IN_TUMOR_MEAN_BORDERLENGTH** | Mean | 0.22 |
| **NUC_IN_TUMOR_MEAN_ROUNDNESS** | Mean | 0.21 |
| **NUC_IN_STROMA_MEAN_BORDERLENGTH** | Mean | 0.19 |
| **NUC_IN_TUMOR_MEAN_AREA** | Mean | 0.19 |
| Collation method across 15 captured images per samples is shown.  Each parameter is ranked by its associated Gini score.  PDC = poorly differentiated clusters. LVI = lymphatic vessel invasion.  Parameters in bold and above the line are the 37 significant parameters reported from the random forest model. | | |
